# Discovery of a new subgroup of sulfur dioxygenases and characterization of sulfur dioxygenases in the sulfur metabolic network of *Acidithiobacillus caldus*

**DOI:** 10.1371/journal.pone.0183668

**Published:** 2017-09-05

**Authors:** Wei Wu, Xin Pang, Jianqiang Lin, Xiangmei Liu, Rui Wang, Jianqun Lin, Linxu Chen

**Affiliations:** State Key Laboratory of Microbial Technology, Shandong University, Jinan, Shandong, China; Universidade Nova de Lisboa, PORTUGAL

## Abstract

*Acidithiobacillus caldus* is a chemolithoautotrophic sulfur-oxidizing bacterium that is widely used for bioleaching processes. *Acidithiobacillus* spp. are suggested to contain sulfur dioxygenases (SDOs) that facilitate sulfur oxidation. In this study, two putative *sdo* genes (A5904_0421 and A5904_1112) were detected in the genome of *A*. *caldus* MTH-04 by BLASTP searching with the previously identified SDO (A5904_0790). We cloned and expressed these genes, and detected the SDO activity of recombinant protein A5904_0421 by a GSH-dependent *in vitro* assay. Phylogenetic analysis indicated that A5904_0421and its homologous SDOs, mainly found in autotrophic bacteria, were distantly related to known SDOs and were categorized as a new subgroup of SDOs. The potential functions of genes A5904_0421 (termed *sdo1*) and A5904_0790 (termed *sdo2*) were investigated by generating three knockout mutants (Δ*sdo1*, Δ*sdo*2 and Δ*sdo1&2*), two *sdo* overexpression strains (OE-*sdo1* and OE-*sdo2*) and two *sdo* complemented strains (Δ*sdo1/sdo1*’ and Δ*sdo2/sdo2*’) of *A*. *caldus* MTH-04. Deletion or overexpression of the *sdo* genes did not obviously affect growth of the bacteria on S^0^, indicating that the SDOs did not play an essential role in the oxidation of extracellular elemental sulfur in *A*. *caldus*. The deletion of *sdo1* resulted in complete inhibition of growth on tetrathionate, slight inhibition of growth on thiosulfate and increased GSH-dependent sulfur oxidation activity on S^0^. Transcriptional analysis revealed a strong correlation between *sdo1* and the tetrathionate intermediate pathway. The deletion of *sdo2* promoted bacterial growth on tetrathionate and thiosulfate, and overexpression of *sdo2* altered gene expression patterns of sulfide:quinone oxidoreductase and rhodanese. Taken together, the results suggest that *sdo1* is essential for the survival of *A*. *caldus* when tetrathionate is used as the sole energy resource, and *sdo2* may also play a role in sulfur metabolism.

## Introduction

*Acidithiobacillus caldus* is an acidophilic, chemolithoautotrophic, sulfur-oxidizing bacterium that is widely used in the bioleaching industry [1, 2, 3]. *A*. *caldus* effectively removes sulfur from the surfaces of minerals, while maintaining acidic conditions that promote the growth of other microorganisms important in the bioleaching process [4]. *A*. *caldus* can oxidize various reduced inorganic sulfur compounds (RISCs), such as tetrathionate (S_4_O_6_^2-^), thiosulfate (S_2_O_3_^2-^), sulfite (SO_3_^2-^), sulfide (S^2-^) and elemental sulfur (S^0^). The ATP and NAD(P)H generated during sulfur oxidation are then used by *A*. *caldus* to fix carbon dioxide and allow autotrophic growth [5, 6]. Given the commercial importance of *A*. *caldus*, the identification of sulfur-oxidizing enzymes can provide important insights into sulfur metabolism in *Acidithiobacillus* spp.

The ability of *Acidithiobacillus* spp. to oxidize sulfur was identified as early as 1959 [7], and identifying the sulfur-oxidizing enzymes has long been a focus of the field. In 1987, a periplasmic sulfur:ferric ion oxidoreductase was purified from *Acidithiobacillus ferrooxidans* and was shown to catalyze the oxidation of S^0^ to sulfite. The enzyme was named sulfur dioxygenase (SDO). The original oxidation experiments were catalyzed in the presence of reduced glutathione (GSH) by using Fe^3+^ or molecular oxygen as the electron acceptor [8]. However, further studies showed that sulfur dioxygenase did not use S^0^ as its substrate, but rather used the sulfane sulfur atom of glutathione persulfide (GSSH) and its higher homologues (GSSnH, n>1), where sulfite was the first product [9, 10].

Although sulfur dioxygenase activity was first reported in *Acidithiobacillus*, the *sdo* genes have been identified only recently [11]. ETHE1 proteins are mitochondrial sulfur dioxygenases in human and *Arabidopsis* that facilitate the catabolism of sulfide in mitochondria along with sulfide:quinone oxidoreductase (SQR) and rhodanese (Rhd) [12, 13, 14]. Comparing the amino acid sequences of these mitochondrial sulfur dioxygenases to heterotrophic bacteria has led to the discovery of three subgroups of SDOs called Blhs, ETHE1s and SdoAs [15]. The 3D structures of an ETHE1 from *Myxococcus xanthus* and a SdoA from *Pseudomonas putida* revealed information of GSH binding and the catalytic mechanisms of these SDOs [16]. An ETHE1 homologue ACAL_0790 (A5904_0790) was found recently in *A*. *caldus* MTH-04 by BLASTP search using the human ETHE1 sequence. Recombinant ACAL_0790 was shown to have sulfur dioxygenase activity *in vitro* [11]. However, whether there are other *sdo* genes in the genome of *A*. *caldus* MTH-04 and whether these putative *sdo* genes have sulfur dioxygenase activity remains unknown.

Here, potential *sdo* genes were searched in the genome of *A*. *caldus* MTH-04. These putative genes were cloned, expressed in *Escherichia coli* and the recombinant proteins were assayed for SDO activity. In addition, the evolutionary relationships among all SDOs were analyzed. Furthermore, the putative *sdo* genes were deleted and complemented, as well as overexpressed, in *A*. *caldus* MTH-04 to test the function of these genes. The enzyme activities of the putative SDO proteins and their effects on cell growth, as well as the expression of sulfur oxidation related genes were characterized. This study provides important information for constructing improved sulfur oxidation models for *Acidithiobacillus* spp., which are important for optimizing bioleaching.

## Materials and methods

### Bioinformatics

SDO homologues were searched in the genome of *A*. *caldus* MTH-04 (CGMCC 1.15711) (GenBank accession number LXQG00000000) using NCBI BLASTP (http://blast.ncbi.nlm.nih.gov/Blast.cgi). The ExPASy Compute pI/Mw tool (http://web.expasy.org/compute_pi/) was used to predict the isoelectric points (pI) and the molecular weights of the homologous proteins. Multiple sequence alignment was performed using ClustalX version 1.81. A neighbor-joining phylogenetic tree of the homologous proteins was constructed with ClustalX version 1.81 and MEGA version 5 with a p-distance distribution, pairwise deletion and bootstrap analysis of 10,000 repeats. Finally, subcellular localizations of proteins were predicted using Softberry ProtCompB tool (http://linux1.softberry.com/berry.phtml?topic=protcompan&group=programs&subgroup=proloc), SignalP 4.1 (http://www.cbs.dtu.dk/services/SignalP/) and PSORTB v3.0 (http://www.psort.org/psortb/).

### Bacterial strains, plasmids, media and growth conditions

Bacterial strains and plasmids used in this study are listed in S1 Table.

*A*. *caldus* was grown at 40°C with shaking at 150 rpm in liquid Starkey-S^0^ medium (pH 2.5), liquid Starkey- K_2_S_4_O_6_ medium (pH 2.5), liquid DMSZ 71- Na_2_S_2_O_3_ medium (pH 4.7) or on solid Starkey-Na_2_S_2_O_3_ medium (pH 4.8) [17, 18]. Liquid Starkey medium contained S^0^ (8 g/L) or K_2_S_4_O_6_ (2.27 g/L) and liquid DMSZ 71 medium contained Na_2_S_2_O_3_·5H_2_O (5 g/L) as the energy source for *A*. *caldus*. K_2_S_4_O_6_ and Na_2_S_2_O_3_ were sterilized by filtration (0.22-μm pore size) and added to the media before inoculation. S^0^ was sterilized by boiling for at least 4 h and was added to the media after inoculation. Kanamycin (200 μg/ml) or chloromycetin (68 μg/ml) were used in liquid Starkey-S^0^ medium, liquid Starkey- K_2_S_4_O_6_ medium or liquid DMSZ 71- Na_2_S_2_O_3_ medium as required, and solid Starkey-Na_2_S_2_O_3_ medium was made with kanamycin (80 μg/ml) or chloromycetin (27.2 μg/ml) for selection. *E*. *coli* strains were grown at 37°C at 170 rpm in liquid Luria-Bertani (LB) broth or on LB agar plates with either ampicillin (100 μg/ml), kanamycin (100 μg/ml), or chloromycetin (34 μg/ml).

### Genetic manipulation

Restriction enzyme digestion, ligation, gel electrophoresis and other general molecular techniques were performed as previously described [19]. *A*. *caldus* genomic DNA was isolated using the TIANamp Bacteria DNA Kit (TIANGEN). Plasmids were isolated using plasmid mini kit I (Omega Bio-Tek). DNA fragments were extracted from agarose gels using a gel extraction kit (Omega Bio-Tek). DNA polymerase, restriction enzymes and T4 DNA ligase were purchased from TaKaRa, and primers were generated by Invitrogen.

### Cloning, expression and purification of SDO homologues

The coding sequences of A5904_0421, A5904_0790 and A5904_1112 were amplified from *A*. *caldus* MTH-04 genomic DNA using PrimeSTAR^®^ HS DNA Polymerase (TaKaRa) with the primer pairs 0421orfF/0421orfR, 0790orfF/0790orfR and 1112orfF/1112orfR (S2 Table), respectively. The fragments were digested with NdeI-XhoI and ligated into NdeI-XhoI treated pET22b(+) to generate the recombinant plasmids pET22b-0421, pET22b-0790 and pET22b-1112. Correct clones were confirmed by sequencing, then the plasmids were transformed into *E*. *coli* BL21(DE3) cells. Expression of recombinant proteins was induced by the addition of 0.4 mM IPTG (isopropyl-β-D-thiogalactopyranoside) at 25°C for 5 h. Cells were collected by centrifugation and washed twice with ice-cold 20 mM NaH_2_PO_4_ buffer (pH 7.4) containing 30 mM imidazole and 500 mM NaCl, and lysed by sonication at 4°C. Supernatants were collected by centrifugation at 13,400×*g* for 30 min at 4°C and the recombinant proteins were analyzed by 10% (wt/vol) SDS-PAGE gels. Proteins were purified using HisTrap^™^ HP Crude columns (GE Health) according to the manufacturer’s instruction. The buffer was exchanged to 50 mM Tris-HCl buffer (pH 7.4), and glycerol was added to a final concentration of 20% before storage at– 20°C. The enzymes were stable for several weeks under the storage conditions. Finally, protein concentrations were measured using the Bradford assay.

### Sulfur dioxygenase activity

Sulfur dioxygenase activity was measured using purified proteins from *E*. *coli* according to a previously published method with minor modifications [11]. Dispersed elemental sulfur suspension was prepared by mixing 32 mg elemental sulfur powder and 5 μl of Tween-20 to 5 ml of 50 mM Tris-HCl buffer (pH 8.0), and sonicating (90 kHz, 15 s on, 5 s off) the mixture for 5 min on ice. The reactional mixture (0.8 ml) contained 0.5 ml dispersed elemental sulfur suspension, 127 μg purified A5904_0421 recombinant protein or 31 μg purified A5904_0790 recombinant protein and GSH was added into the reaction mixture to a final concentration of 0.8 mM before measurement. The assays were performed at 40°C with shaking at 150 rpm for 30 min. All samples were then centrifuged for 5 min at 13,400 × *g* and 4°C to remove remaining sulfur, and the supernatant was used to measure the amount of sulfite produced during the reaction. The amount of sulfite was measured according to a previously published method [20]. Heat inactivated purified proteins were used as controls for each experiment. When the activity was calculated, the sulfite produced by heat inactivated proteins was deducted. One unit of SDO activity was defined as the production of 1 μmol of sulfite per minute. Specific SDO activity was measured as units per milligram of protein.

### pH and temperature optima

The influence of pH and temperature on the SDO activity of the purified recombinant proteins was measured using the method described above. SDO activity over a range of pH values was measured at 40°C in 50 mM citric acid/NaH_2_PO_4_ buffer (pH 3.0–6.0), 50 mM Tris/HCl buffer (pH 7.0–8.0) or 50 mM glycine/NaOH buffer (pH 9.0–10.0). The influence of temperature was measured at pH 8.0 over the range 30–70°C.

### Inhibition studies

The influence of metal ions (Mg^2+^, Mn^2+^, Fe^3+^, Ni^2+^, Zn^2+^, Cu^2+^, Co^2+^ and Hg^2+^), EDTA, DTT and N-ethylmaleimide (NEM) on SDO activity was evaluated by performing the SDO activity assay as described above in the presence of each reagent in 50 mM Tris/HCl buffer (pH 8.0) at 40°C.

### Kinetic analysis

Kinetic analysis was carried out in 50 mM Tris/HCl buffer (pH 8.0) at 45°C with 127 μg purified A5904_0421 recombinant protein or 31 μg purified A5904_0790 recombinant protein and various concentrations of GSSH (range over 95.3–953 μM). GSSH was prepared by mixing equal volumes of 17 mM glutathione in 50 mM Tris/HCl buffer (pH 8.0) with a saturated sulfur solution, containing 17 mM elemental sulfur. The GSSH concentration was determined according to a previously published method [21]. The initial reaction rate was determined for each GSSH concentration. *K*_m_ values were determined by nonlinear regression analysis of the initial reaction rates against GSSH concentrations fitted to the Michaelis–Menten equation using the software program Origin 8.0 (Originlab Software).

### Total GSH-dependent sulfur oxidation activity assays of *A*. *caldus*

The total GSH-dependent sulfur oxidation activity of *A*. *caldus* was measured using *A*. *caldus* cell extracts according to the method described for sulfur dioxygenase activity. *A*. *caldus* strains were sonicated on ice for 10 min, centrifuged for 5 min at 13,400 × *g* and the supernatant was removed and used as the cell extract for the GSH-dependent sulfur oxidation activity assays. The assay was performed at 40°C in 50 mM Tris/HCl buffer (pH 8.0). Heat inactivated *A*. *caldus* cell extracts were used as controls for each experiment. One unit of the total GSH-dependent sulfur oxidation activity was defined as the production of 1 μmol of sulfite per minute. The total GSH-dependent sulfur oxidation activity was measured as units per milligram of protein.

### Generation of the markerless *sdo* knockout mutant of *A*. *caldus*

Markerless *sdo* knockout mutants were generated as described previously with minor modifications [22]. All primers used in this section are listed in S2 Table. The suicide plasmids pSDUDI-*sdo1* (specific to A5904_0421) and pSDUDI-*sdo2* (specific to A5904_0790) were made using pSDUDI as the plasmid backbone. The upstream and downstream homologous arms of *sdo1* were amplified using the primer pair *sdo1*UF/*sdo1*UR to amplify the 1,211 bp upstream homologous arm, and primers *sdo1*DF/*sdo1*DR to amplify the 722 bp downstream homologous arm. The homologous arms of *sdo2* were amplified using primer pair *sdo2*UF/*sdo2*UR to amplify the 1,109 bp upstream homologous arm and primers *sdo2*DF/*sdo2*DR to amplify the 1,275 bp downstream homologous arm. The sequences were ligated with pSDUDI after digestion with appropriate restriction enzymes. Successful insertions were confirmed by sequencing, and the resulting recombinant plasmids were named pSDUDI-*sdo1* and pSDUDI-*sdo2*.

pSDUDI-*sdo1* or pSDUDI-*sdo2* was transformed into *E*. *coli* SM10 by selecting for ampicillin resistant clones and these transformants were used as donor strains for conjugation. *A*. *caldus* MTH-04 was used as the recipient to generate the *A*. *caldus* Δ*sdo1* or Δ*sdo2* strains. Δ*sdo1* was used as the recipient to construct the Δ*sdo1&2* strain. pSDUDI-*sdo1* or pSDUDI-*sdo2* was transferred from *E*. *coli* SM10 to *A*. *caldus* by conjugation. Both donor cells and recipient cells were cultivated until exponential phase, collected by centrifugation, and mixed at a ratio of approximately 1:3, then spotted onto a 25 mm diameter, 0.22-μm pore size sterilized filter membrane that was placed on the mating medium. After incubation at 37°C for 3 days, cells on the filter membrane were suspended in ddH_2_O and plated on selective Starkey-Na_2_S_2_O_3_ plates containing kanamycin. Colonies were selected and analyzed by colony PCR using primer pairs located in the homologous arms to amplify a partial sequence of the homologous arms. Primer pair *sdo1*inF*/sdo1*inR was used to detect single crossovers of Δ*sdo1*, where 1,906 bp and 1,168 bp fragments were detected when a single crossover had occurred. Primer pair *sdo2*inF/*sdo2*inR was used to detect the single crossover of Δ*sdo2* or Δ*sdo1&2*, where 1,721 bp and 974 bp fragments were detected in the case of a single crossover. Colonies with the correct PCR fragments were inoculated into liquid Starkey-S^0^ medium and the genomic DNA of each selected colony was isolated for PCR analysis to further confirm the single crossover-recombination event.

Plasmid pSDU1-I-Sce I was introduced into the single-crossover cells of *A*. *caldus* to express I-SceI endonuclease to digest its recognition site (5’-TAGGGATAACAGGGTAAT-3’) that was introduced by the suicide plasmid. This creates double-stranded DNA breaks (DSBs) on the chromosome to induce the second homologous recombination event, generating knockout mutants or wild type individuals. The colonies grown on selective Starkey-Na_2_S_2_O_3_ plates containing chloromycetin were tested for genetic changes by colony PCR as described above. *sdo1*inF/*sdo1*inR primers were used to detect Δ*sdo1* or Δ*sdo1&2*. *sdo2*inF/*sdo2*inR primers were used to detect Δ*sdo2* or Δ*sdo1&2* (predicted sizes of the fragments amplified by PCR were listed in S3 Table). *Sdo* gene knockout mutants were cultured in liquid Starkey-S^0^ medium at 40°C. The genomic DNA of each colony was isolated for PCR analysis to confirm *sdo* gene knockout mutants. Primer pairs 0421orfF/0421orfR, *sdo1*inF/*sdo1*inR and *sdo1*outF/*sdo1*outR were used to confirm the *sdo1* deletion, and primer pairs 0790orfF/0790orfR, *sdo2*inF/*sdo2*inR and *sdo2*outF/*sdo2*outR were used to confirm the *sdo2* deletion (S3 Table, S1 Fig). Primer pairs *sdo1*outF/*sdo1*outR and *sdo2*outF/*sdo2*outR were located outside the homologous arms. The amplified PCR products from the mutants using primer pairs of *sdo1*outF/*sdo1*outR and *sdo2*outF/*sdo2*outR were sequenced to confirm the mutants.

The pSDU1-I-Sce I plasmid in the mutant cells was eliminated by propagating the Δ*sdo* mutants 5 to 10 times in liquid S^0^ medium without chloromycetin, and plating the cells on non-selective solid Starkey-Na_2_S_2_O_3_ medium. Colony PCR was carried out to verify the loss of the plasmid using the primer pairs pSDU1cxs-pSDU1cxa. No fragment was expected if pSDU1-I-Sce I had been lost.

### Construction of *A*. *caldus sdo* overexpression strains and *sdo* complemented strains

The coding sequence of *sdo1* and *sdo2* were amplified from *A*. *caldus* MTH-04 genomic DNA using *sdo1*F/*sdo1*R and *sdo2*F/*sdo2*R primer pairs, respectively (S2 Table). Sequences were digested with restriction enzymes and inserted downstream of the *tac* promoter in the plasmid pSDU1-tac to produce pSDU1-*sdo1* and pSDU1-*sdo2*, and the insertions were confirmed by sequencing. The original plasmid pSDU1 was used as a control. The transfer of plasmids from *E*. *coli* SM10 into *A*. *caldus* MTH-04 or Δ*sdo* strains was performed by conjugation as described above.

### Growth measurements of *A*. *caldus* strains

The *A*. *caldus* MTH-04 wild type, *sdo* knockout mutants, control strain (wild type carrying plasmid pSDU1), *sdo* overexpression strains, and *sdo* complemented strains were grown in Starkey-S^0^ medium for 7 days and cells were collected by centrifugation and adjusted to the same cell density (OD_600_ = 20.0). A 50 μl aliquot of the treated cells was inoculated into 150 ml fresh Starkey-S^0^ medium, Starkey- K_2_S_4_O_6_ medium or DMSZ 71- Na_2_S_2_O_3_ medium. *A*. *caldus* growth in Starkey-S^0^ medium or Starkey- K_2_S_4_O_6_ medium was monitored by measuring the optical density at 600 nm after low-speed centrifugation at 400 × *g* for 5 min, according to the previous report of Wang *et al*. [22]. Growth of *A*. *caldus* in DMSZ 71- Na_2_S_2_O_3_ medium was monitored by cell counting. In order to determine the dry weight cell yield, wild type *A*. *caldus* MTH-04 cells grown to stationary growth phase on the energy source of tetrathionate were collected by centrifugation (5,000 × *g*, 5 min) and dried to a constant weight. The consumption of K_2_S_4_O_6_ was determined by high performance liquid chromatography (HPLC, UL-3000 system; Dionex) equipped with a UV detector, according to a previously published method with minor modifications [23]. The HPLC employed a C18 column (250×4.6 mm, inner diameter, 5 μm; Sepax) with a flow-rate of 0.6 ml per min and the detector was set at 230 nm. The mobile phase consisted of 20% (vol/vol) acetonitrile and 80% (vol/vol) water (containing 6 mM tetrapropylammoniumhydroxide, pH 5.0). The injection volume was 10 μl. The production of sulfate and the consumption of Na_2_S_2_O_3_ were determined by ion chromatography (ICS-1100 system; Dionex) with a mobile phase that contained 25 mM KOH at a flow rate of 1 ml per min. All measurements were performed in triplicate from at least three independent biological replicates. The *P*-value was calculated using a *t*-test.

### RNA extraction and RT-qPCR

To measure relative RNA transcript levels of the genes involved in sulfur metabolism, *A*. *caldus* strains were grown in Starkey-S^0^ medium or Starkey- K_2_S_4_O_6_ medium, collected by centrifugation at 12,000 × *g* for 5 min, resuspended in RNA*later*^®^ Solution (Ambion), stored overnight at 4°C and harvested from the RNA*later*^®^ suspension by centrifugation. Cells were resuspended in100 μl of lysis buffer (1 mg/ml lysozyme, 10 mM Tris and 1 mM EDTA, pH 8.0) and incubated at 26°C for 6 min. Total RNA was extracted using Trizol^®^ reagent (Ambion) according to the manufacturer’s instruction. RNA quality was determined by denaturing formaldehyde agarose gel electrophoresis and RNA concentration was determined using a NanoDrop-1000 spectrophotometer (NanoDrop Technologies). Genomic DNA was removed using the PrimeScript^™^ RT reagent kit with gDNA Eraser (Perfect Real Time)(TaKaRa) and confirmed by PCR using primer pair *alaS*F/*alaS*R (S4 Table). cDNA was synthesized using the PrimeScript^™^ RT reagent kit with gDNA Eraser (Perfect Real Time)(TaKaRa).

RT-qPCR reactions were performed on a LightCycler^®^ 480 system (Roche) with SYBR^®^Premix Ex Taq (TaKaRa), according to the manufacturer’s instructions. All RT-qPCR reactions in this study were performed in technical triplicates from at least three independent biological replicates. Primers used for gene expression analysis are listed in S4 Table. Changes in the relative RNA transcript levels of the sulfur-oxidizing genes in the wild type, knockout mutants and overexpression strains were normalized against the internal control *alaS*, and calculated using the comparative ΔΔC_T_ method and shown as 2^-ΔΔCT^ [24]. Genes showing a fold change ≧2 or ≦0.5 and *P* < 0.05 were considered to be differentially expressed. The *P*-value was calculated using a *t*-test.

### Sequence accession numbers

The genome of *A*. *caldus* MTH-04 (CGMCC 1.15711) has been deposited in the NCBI GenBank database under the accession number LXQG00000000. A5904_0421, A5904_0790 and A5904_1112 sequences have been deposited in the NCBI GenBank database under accession numbers OAN04050.1, OAN03714.1 and OAN03376.1, respectively.

## Results

### Discovery and identification of SDOs in *A*. *caldus* MTH-04

A BLASTP search was conducted using the previously identified SDO (A5904_0790) [11] against the *A*. *caldus* MTH-04 draft genome, which resulted in the identification of two more putative *sdo* genes, A5904_0421 and A5904_1112. The ORF of A5904_0421 shares 33% amino acid identity with A5904_0790, and encodes a 245 amino acid metallo-β-lactamase with a theoretical isoelectric point of 5.02 and molecular mass of 26.2 kDa. The ORF of A5904_1112 is 34% identical to A5904_0790, and encodes a 243 amino acid metallo-β-lactamase with a theoretical isoelectric point of 7.09 and molecular mass of 27.0 kDa (S2 Fig).

**Fig 1:**
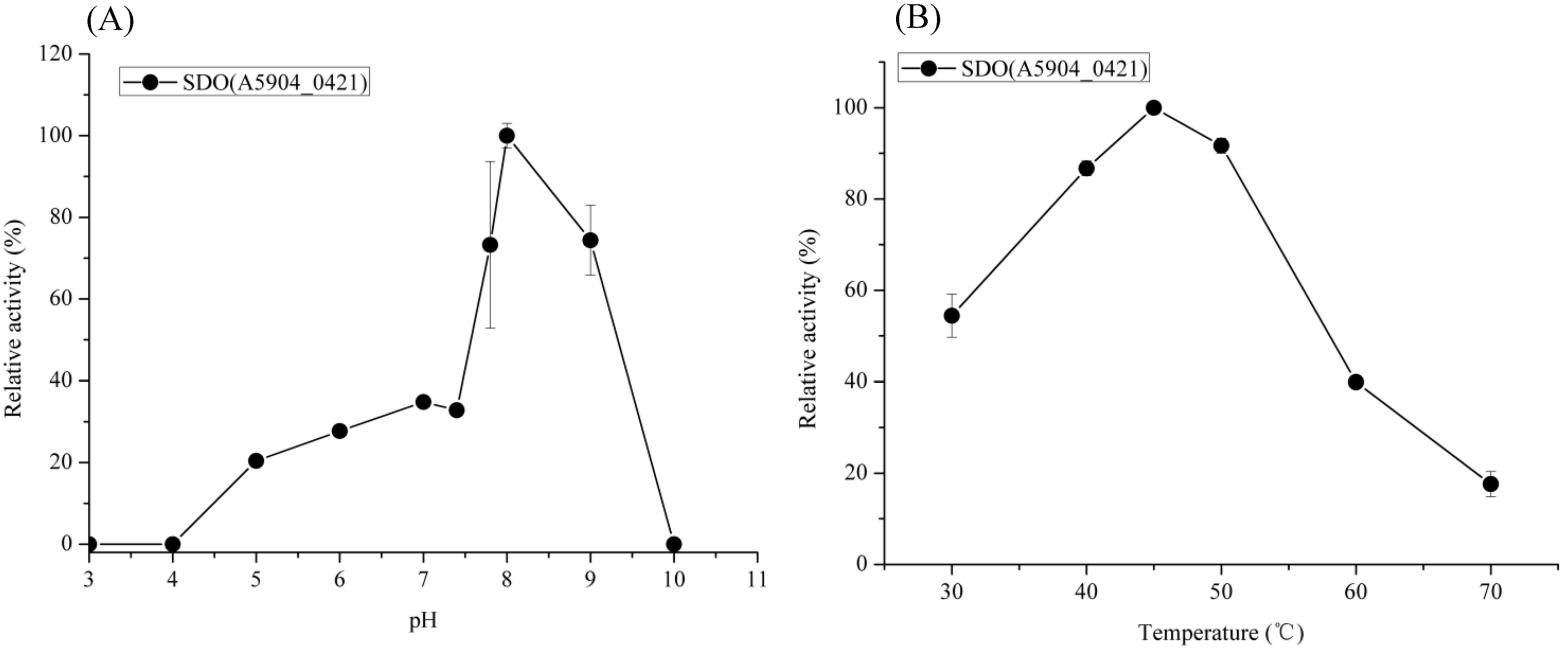
Influence of pH (A) and temperature (B) on SDO activities of purified recombinant A5904_0421. The values corresponding to the 100% activity are 57 mU/mg in (A) and 66 mU/mg in (B), respectively.

A GSH-dependent *in vitro* SDO activity assay was conducted using recombinant proteins A5904_0421, A5904_0790 and A5904_1112 that had been expressed and purified from *E*. *coli*. SDO activity was not detected between pH 3 and pH 10 for A5904_1112. However, SDO activity was detected between pH 5.0 and 9.0 for A5904_0421, with an optimum at pH 8.0 (Fig 1A). The temperature profile of the purified A5904_0421 was determined at pH 8.0 and showed that this protein exhibited optimal activity between 45°C and 50°C (Fig 1B). Based on the *in vitro* SDO activity, A5904_0421 was designated as SDO1 and A5904_0790 was designated as SDO2.

The kinetic parameters of the SDOs were determined in 50 mM Tris/HCl buffer (pH 8.0) at 45°C. The *K*_m_ of SDO1 for GSSH was 267±31 μM and *k*_cat_ was 5.4 s^-1^. The *K*_m_ of SDO2 for GSSH was 298±13 μM and *k*_cat_ was 48.1 s^-1^.There was no significant difference between the *K*m values of SDO1 and SDO2 (P>0.05). The *k*_cat_/*K*_m_ of SDO1 and SDO2 were 20.2 and 161.4 mM ^-1^s^-1^, respectively. SDO2 had the highest catalytic efficiency of the two enzymes according to the *k*_cat_/*K*_m_ ratio (Table 1). The *K*_m_ values of SDO1 and SDO2 were larger than that of *Urechis unicinctus* ETHE1 (82.5 μM [21]), smaller than that of human ETHE1 (340 μM [14]) and within the range of those of the bacterial SDOs (108 μM– 342 μM [15]).

**Table 1:** Kinetic parameters of SDOs.

SDO	*k*_cat_ (s^-1^)	*K*_m_ (μM)	*k*_cat_/*K*_m_ (mM ^-1^s^-1^)
SDO1	5.4	267±31	20.2
SDO2	48.1	298±13	161.4

The influence of metal ions on the SDO activity of SDO1 was also determined (S5 Table). Ni^2+^, Cu^2+^, Co^2+^ and Hg^2+^ were found to strongly inhibit the activity of SDO1, while SDO activity was inhibited to some extent by Mg^2+^, Zn^2+^, Mn^2+^ and Fe^3+^. The divalent metal chelator EDTA completely inhibited the SDO activity of SDO1, indicating that a divalent metal ion might be essential for the activity of this enzyme. 0.05 mM DTT did not significantly affect SDO activity while the thiol modifying reagent NEM strongly inhibited SDO activity, suggesting that free SH group(s) are needed for the catalytic activity of SDO1, as previously reported for other SDOs [11].

### Distribution of SDOs in *Acidithiobacillus* spp and other sulfur oxidizers

**Fig 2:**
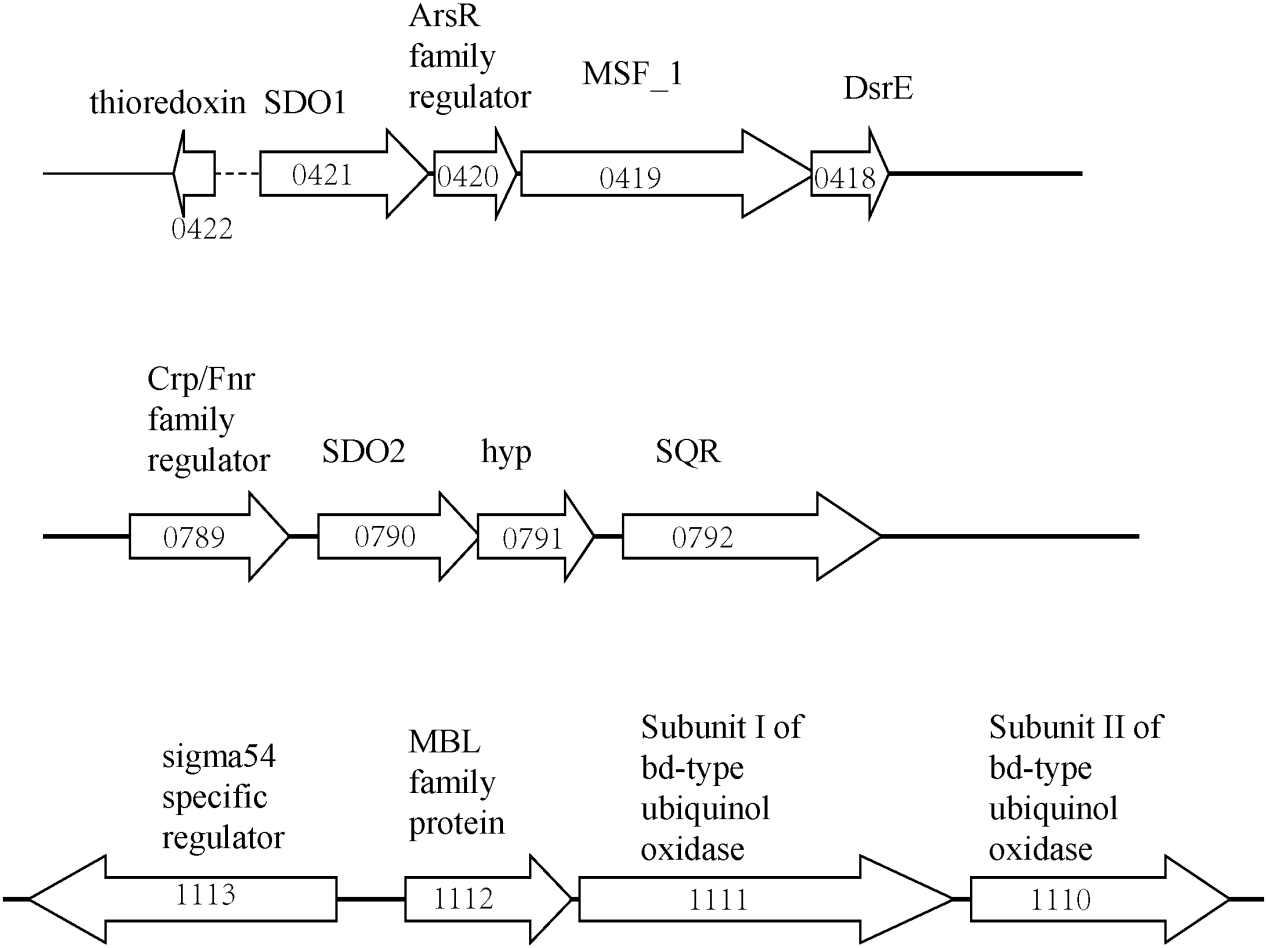
Genes co-localized with SDOs in *A*. *caldus* MTH-04. The predicted functions of genes are shown. The locus-tags are given inside of the gene arrows.

BLASTP analysis of bacterial and archaeal genomes using the SDO1 or SDO2 amino acid sequences as a query revealed that SDO homologues are widespread in *Acidithiobacillus* spp., and there were two to three copies of SDO in different strains of *Acidithiobacillus* spp. bacteria (Table 2). The *sdo* genes in *A*. *caldus* MTH-04 are co-localized with other genes related to sulfur-metabolism. A *dsrE*-like gene (A5904_0418), which potentially encodes a protein involved in sulfur transfer, is downstream of *sdo1*. A *sqr-*like gene (A5904_0792) is downstream of *sdo2*. A5904_1112 (with no *in vitro* SDO activity) is not co-localized with genes related to sulfur-metabolism (Fig 2).

**Table 2:** Amino-acid sequence identities of the sulfur dioxygenases between *A*. *caldus* MTH-04 and other *Acidithiobacillus* bacteria.

Strain	GenBank accession number	subgroup	aa identity (%)	
			A5904_0421	A5904_0790
*A*. *caldus* ATCC 51756	AIA56289.1	ETHE1		99
	AIA55587.1	SdoS	98	
*A*. *caldus* SM-1	AEK59246.1	ETHE1		100
	AEK57201.1	SdoS	100	
	AEK58550.1	SdoS	100	
*A*. *ferroxidans* ATCC 23270	ACK79351	ETHE1		73
	ACK80268.1	ETHE1		51
*A*. *thiooxidans* ATCC 19377	WP_010638935.1	ETHE1		72
	WP_010641840.1	SdoS	84	
*A*. *thiooxidans* A01	WP_024894467.1	SdoS	82	
	WP_024893175.1	SdoA		34
	WP_024895058.1	SdoS	84	
*A*. *ferrivorans* SS3	WP_014028753.1	SdoS	80	
	WP_014029574.1	ETHE1		51

**Fig 3:**
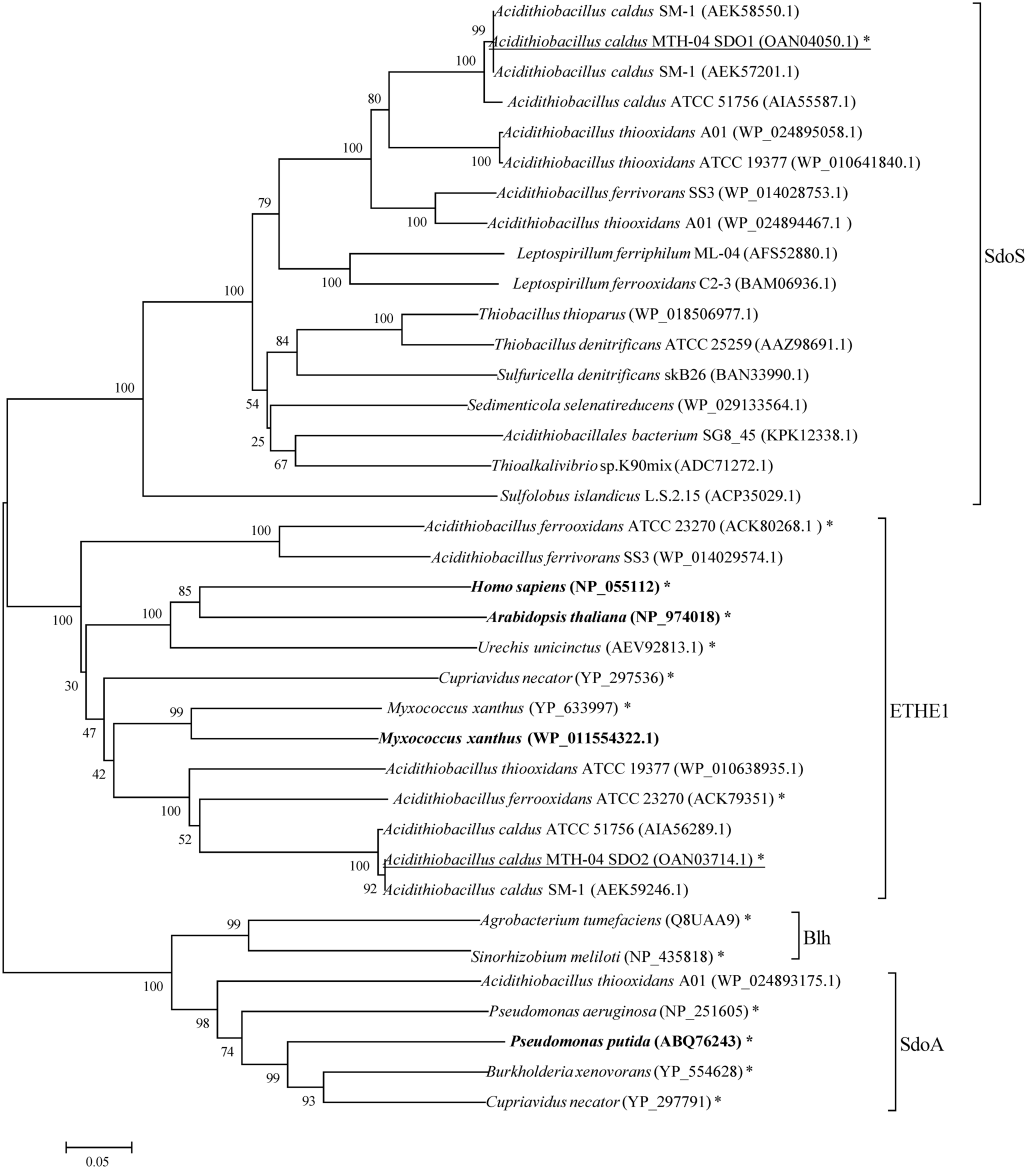
A neighbor-joining tree of SDOs from eukaryotic and prokaryotic species. GenBank accession numbers are presented in the parentheses. The proteins with SDO activities demonstrated in this study or previous studies are marked by asterisk (*). The SDOs with known 3D structures are highlighted in bold. The proteins from *A*. *caldus* MTH-04 are underlined.

A phylogenetic analysis was conducted to better understand the relationship between previously described SDOs (ETHE1s, Blhs, SdoAs) [15] and the putative SDOs detected in *Acidithiobacillus* spp. and other sulfur oxidizing bacteria (Fig 3). *A*. *caldus* SDO2 showed a closer evolutionary relationship to known ETHE1s from the mitochondria of eukaryotes, while *A*. *caldus* SDO1 and its homologues, mainly found in autotrophic bacteria, were distantly related to other known SDOs. A new group, containing SDO1 from *A*. *caldus* MTH-04, and SDOs from autotrophic sulfur-oxidizing bacteria, such as *Acidithiobacillus* spp., *Thiobacillus* spp., *Acidithiobacillales* sp., *Sulfuricella* sp., *Thioalkalivibrio* sp., was designated as subgroup SdoS.

### Construction and characterization of *sdo* knockout mutants, *sdo* overexpression strains and *sdo* complemented strains of *A*. *caldus* MTH-04

To further study the function of the SDOs in *A*. *caldus*, a markerless gene knockout system was used to generate the *sdo* single knockout strains Δ*sdo1* and Δ*sdo2*, as well as the *sdo* double knockout strain Δ*sdo1&2*. The complete sequences of *sdo1* and *sdo2* were deleted in the knockout strains and the mutants were identified by PCR using different primer sets (S1 Fig). All observed PCR fragments were in accordance with the predicted sizes as listed in S3 Table. The correct sequences of the mutants were confirmed by sequencing the mutated regions. The *sdo* overexpression strains (OE-*sdo1* and OE-*sdo2*), *sdo* complemented strains (Δ*sdo1/sdo1*’ and Δ*sdo2/sdo2*’) and a control strain (wild type carrying plasmid pSDU1) were generated as described in the Materials and Methods section.

**Fig 4:**
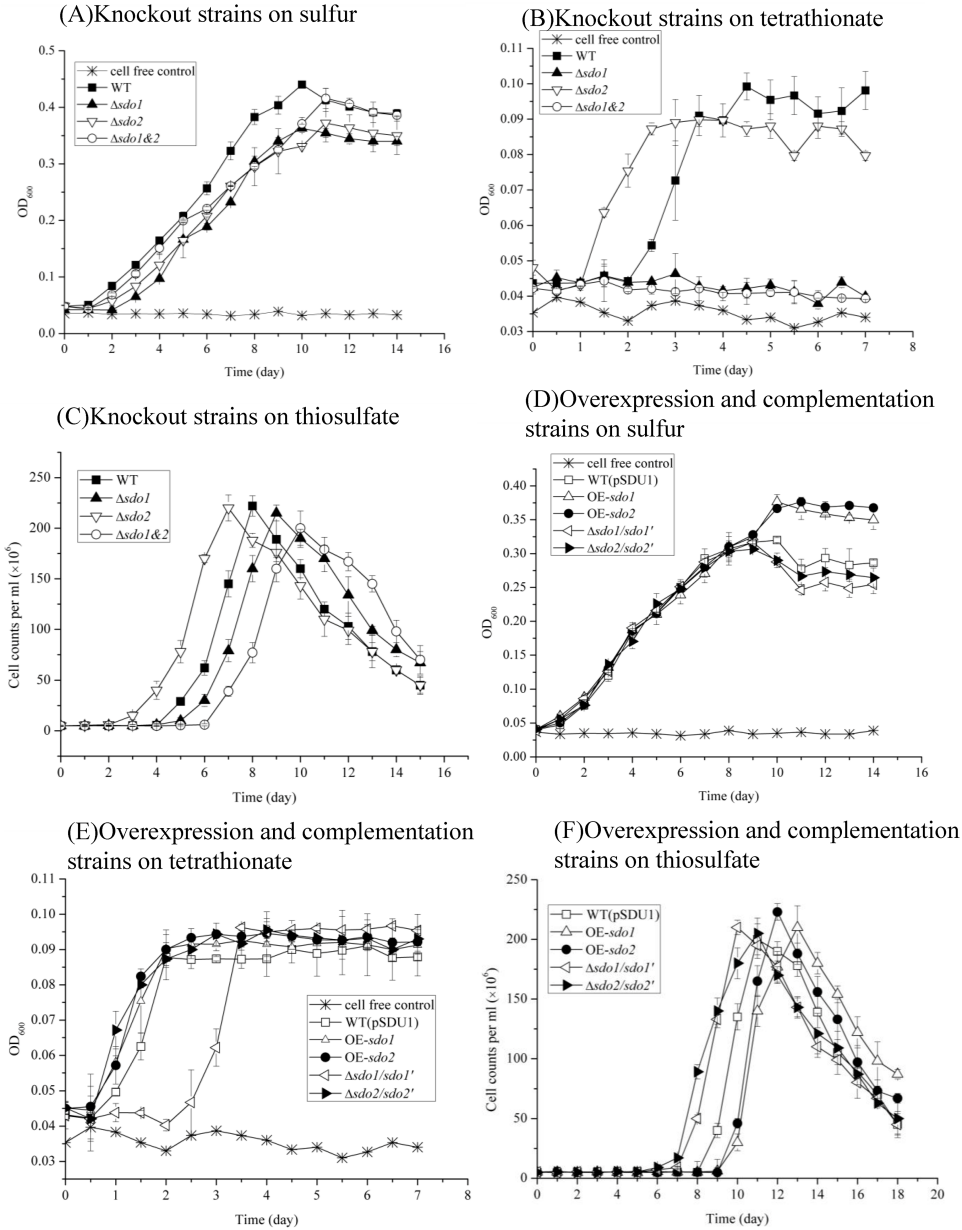
Growth curves of the *A*. *caldus* MTH-04 wild type, *sdo* knockout mutants, *sdo* overexpression strains, *sdo* complemented strains and control strain (wild type carrying pSDU1) grown in S^0^ medium, tetrathionate medium or thiosulfate medium. (A-C) Growth of *sdo* knockout mutants compared with wild type on S^0^ (A), tetrathionate (B) and thiosulfate (C). (D-F) Growth of *sdo* overexpression strains and *sdo* complemented strains compared with the control strain (wild type carrying pSDU1) on S^0^ (D), tetrathionate (E) and thiosulfate (F). WT represent the wild type, Δ*sdo1*, Δ*sdo2*, Δ*sdo1&2* represent the *sdo* knockout mutants, WT (pSDU1) represent the control strain, OE-*sdo1* and OE-*sdo2* represent the *sdo* overexpression strains, Δ*sdo1/sdo1’* and Δ*sdo2/sdo2’* represent the *sdo* complemented strains of *A*. *caldus* MTH-04, respectively. OD_600_ indicates the optical density at 600 nm, all measurements were performed in triplicate and error bars correspond to the standard deviations.

To understand the effect of *sdo* genes on the growth of *A*. *caldus*, wild type and recombinant *A*. *caldus* strains (including knockout, complemented and overexpression strains) were grown in Starkey-S^0^ medium, Starkey- K_2_S_4_O_6_ medium and DMSZ71- Na_2_S_2_O_3_ medium. In general, the Δ*sdo1*, Δ*sdo2* and Δ*sdo1&2* strains only showed slight differences in growth when compared to wild type on S^0^. Δ*sdo1* had a slightly reduced growth rate compared to the wild type (P<0.05), and Δ*sdo2* had a similar growth rate to Δ*sdo1* (P>0.05), while Δ*sdo1&2* had a slight growth advantage over Δ*sdo1*and Δ*sdo2* during the first 5 days (P<0.05) (Fig 4A). Δ*sdo1* and Δ*sdo1&2* did not survive on tetrathionate. Interestingly, Δ*sdo2* entered the logarithmic growth phase one day earlier than wild type when grown on tetrathionate (Fig 4B). The complemented *sdo1* strain rescued the wild-type phenotype when grown on tetrathionate (Fig 4E). These results suggested that *A*. *caldus* requires *sdo1*, but not *sdo2*, to grow on tetrathionate. When thiosulfate was used as the energy source, Δ*sdo2* still entered the logarithmic growth phase one day earlier than wild type. Δ*sdo1* and Δ*sdo1&2* showed a slightly reduced growth capacity compared to wild type (P<0.05) (Fig 4C). Overexpression of *sdo* genes did not increase the growth rate compared to the control strain (wild type carrying plasmid pSDU1) on S^0^ (Fig 4D), tetrathionate (Fig 4E) or thiosulfate (Fig 4F).

**Fig 5:**
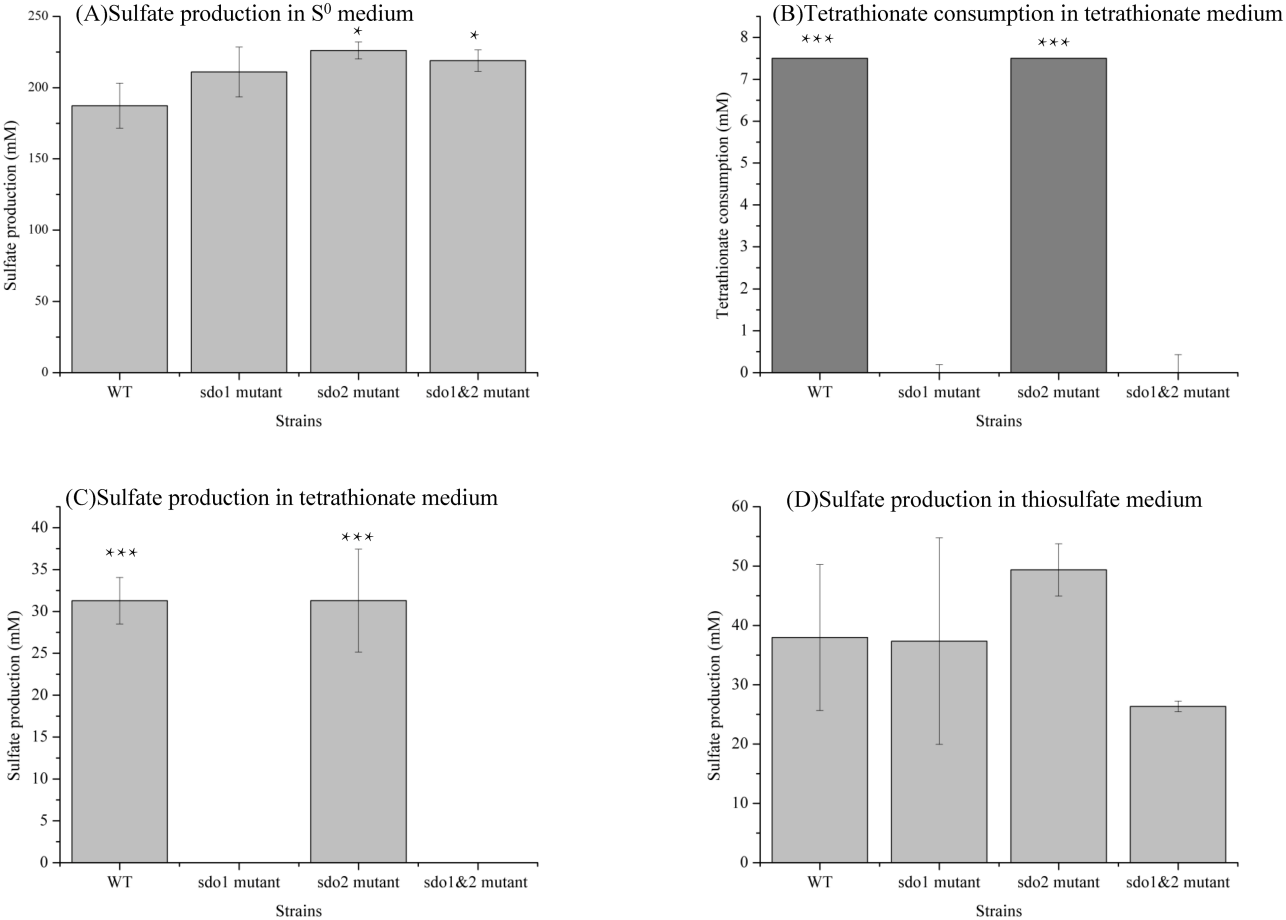
Comparison of sulfate production and tetrathionate consumption of the *A*. *caldus* MTH-04 wild type and *sdo* knockout mutants grown in S^0^ medium, tetrathionate medium and thiosulfate medium. (A) Sulfate production in S^0^ medium. (B) Tetrathionate consumption in tetrathionate medium. (C) Sulfate production in tetrathionate medium. (D) Sulfate production in thiosulfate medium. WT represent the wild type, *sdo1* mutant, *sdo2* mutant, *sdo1&2* mutant represent the *sdo* knockout mutants. At least three independent biological replicates were tested and error bars correspond to the standard deviations. Asterisks denote statistically significant changes (*P < 0.05, *** P < 0.001).

When S^0^ was used as the energy source, sulfate was the only detected product, and the knockout mutants produced more sulfate (Fig 5A) but grew less than the wild type (Fig 4A). This result indicated that the wild type grew more efficiently as it had a complete metabolic network. When grown on tetrathionate, the wild type and Δ*sdo2* strains used all the 7.5 mM tetrathionate (Fig 5B) to produce an equal amount of sulfate (approximately 31 mM) (Fig 5C). No tetrathionate was consumed by the Δ*sdo1* and Δ*sdo1&2* strains (Fig 5B), which was in agreement with lack of observed growth (Fig 4B). When thiosulfate was used as the energy source, no thiosulfate was detected in the end and sulfate was the only detected product. Na_2_S_2_O_3_ easily degrades into S^0^ and SO_3_^2-^ at pH lower than 4.4, and the produced S^0^ could be used by *A*. *caldus* strains as well. As the pH decreased soon after the growth began, degradation might contribute to the exhaustion of thiosulfate as well. There was no significant difference in sulfate production among wild type and *sdo* knockout mutants (*P*>0.05)(Fig 5D).

### Analysis of the total GSH-dependent sulfur oxidation activity of *A*. *caldus*

**Fig 6:**
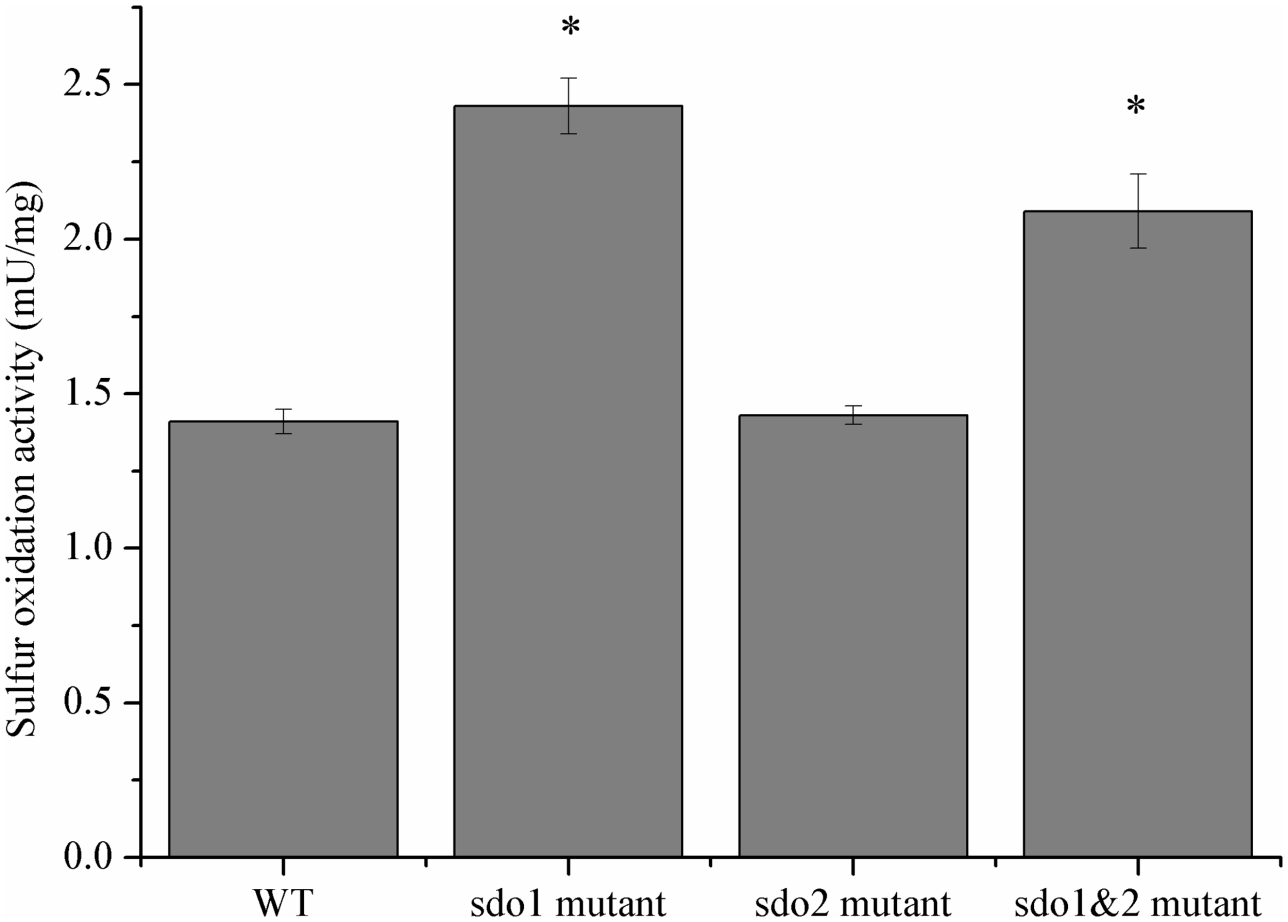
Sulfur oxidation activity levels (mU/mg) in the cell extracts from wild type (WT) and mutant strains of *A*. *caldus* MTH-04 grown on S^0^. Asterisks denote statistically significant changes (*P < 0.05).

We tested the sulfur oxidation activity of the Δ*sdo* strains to study the effect of *sdo* gene deletion on the sulfur metabolic network of *A*. *caldus*. The total GSH-dependent sulfur oxidation activities of the wild type and mutant *A*. *caldus* strains grown in Starkey-S^0^ medium were measured using the cell extracts of these strains. As shown in Fig 6, the GSH-dependent sulfur oxidation activities of Δ*sdo1* and Δ*sdo1*Δ*sdo2* increased 72% and 48%, respectively, compared to the wild type, while the activity of the Δ*sdo2* strain was approximately the same as that of the wild type (Fig 6). The high growth rates of *sdo* single- and double-knockout mutants in Starkey-S^0^ medium and the high level of GSH-dependent sulfur oxidation activity in these mutants, indicated that there may be undetected S^0^-oxidizing enzymes in *A*. *caldus*.

### Transcriptional analysis of *A*.*caldus* strains

**Fig 7:**
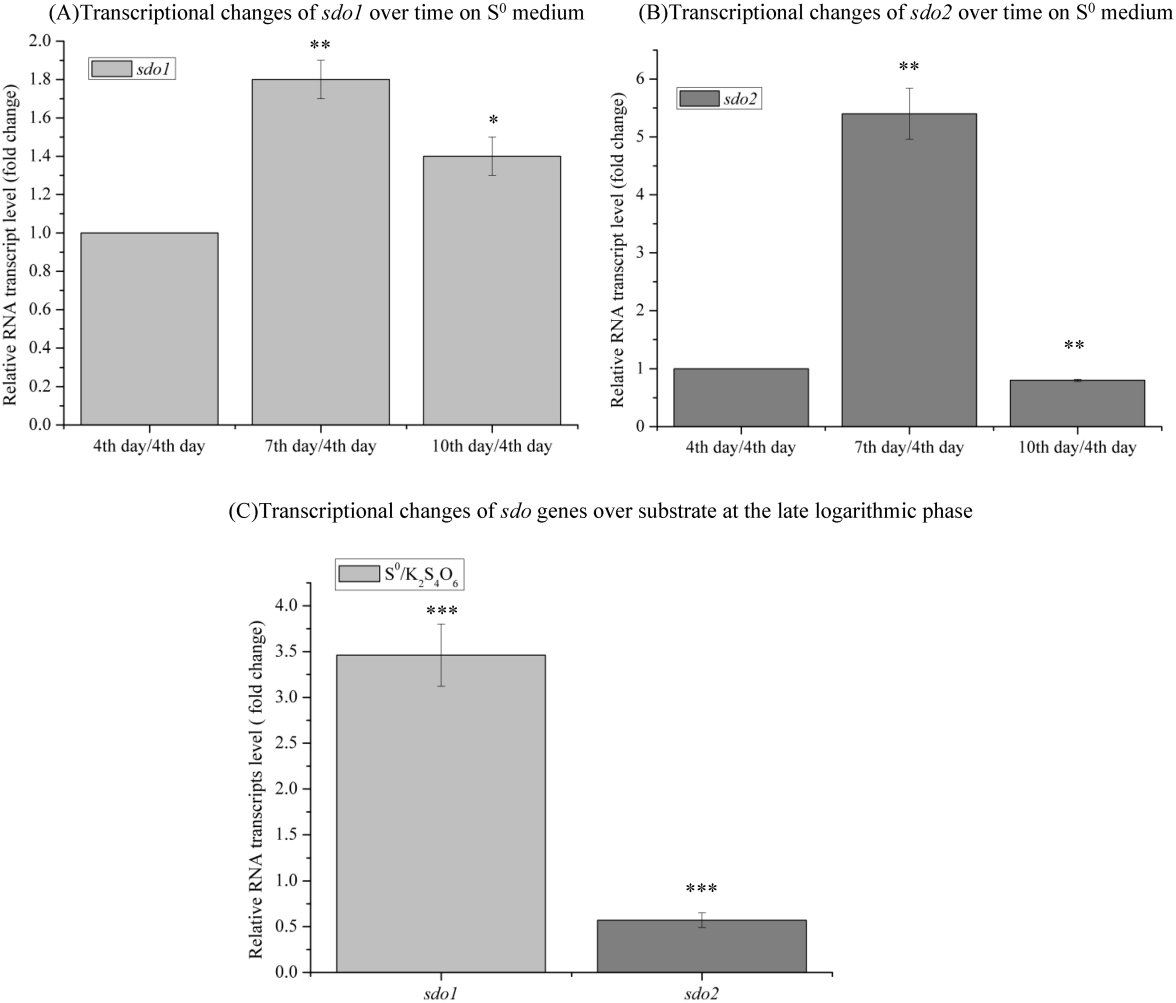
Relative RNA transcript level changes of *sdo* genes in *A*. *caldus* MTH-04 wild type in S^0^ medium or tetrathionate medium. (A-B) RNA transcript level of *sdo1* (A) and *sdo2* (B) on the 7th (late logarithmic phase) and 10th (early stationary phase) days compared with that of the 4th day (early logarithmic phase) in S^0^ medium. (C) RNA transcript level of *sdo* genes in S^0^ medium compared to that in tetrathionate medium at the late logarithmic phase. Asterisks denote statistically significant changes (*P < 0.05, **P < 0.01, *** P < 0.001).

Cells of the wild type *A*. *caldus* grown on S^0^ were collected on the 4th (early logarithmic phase), 7th (late logarithmic phase) and 10th (early stationary phase) days of growth (Fig 4A) to purify total RNA for transcriptional analysis. Wild type *A*. *caldus* cells grown on tetrathionate were collected on the 2nd day (late logarithmic phase) (Fig 4B) to purify total RNA for transcriptional analysis. When S^0^ was used as the sole energy source, the RNA transcript level of *sdo1* on the 7th (late logarithmic phase) and 10th (early stationary phase) days were 1.8-fold and 1.4-fold, respectively, compared with that of the 4th day (early logarithmic phase) (P<0.05) (Fig 7A), while the RNA transcript level of *sdo2* on the 7th (late logarithmic phase) and 10th (early stationary phase) days were 5.4-fold and 0.8-fold, respectively, compared with that of the 4th day (early logarithmic phase) (P<0.01) (Fig 7B). These results showed that *sdo* genes were highly transcribed in the late logarithmic growth phase on S^0^. In the late logarithmic phase, the RNA transcript level of *sdo1* on S^0^ medium increased to 3.5-fold compared with that on tetrathionate medium, and the RNA transcript level of *sdo2* on S^0^ medium decreased to 0.6-fold compared with that on tetrathionate medium (P<0.001) (Fig 7C). This suggests that the metabolic substrates available to *A*. *caldus* significantly influenced *sdo1* expression.

To further understand the influence of deletion or overexpression of *sdo* genes on other sulfur-oxidizing genes in *A*. *caldus*, the relative mRNA levels in wild type, mutants, overexpression strains and control strain (wild type carrying plasmid pSDU1) were measured using RT-qPCR. Cells were grown to late logarithmic phase either with S^0^ or tetrathionate as the sole energy source. Relative RNA transcript levels of these genes were determined against the wild type for *sdo* knockout strains, or the control strain (wild type carrying pSDU1) for *sdo* overexpression strains. When S^0^ was used as the sole energy, the RNA transcript levels of *tetH* and *tqo* were increased in *sdo* knockout strains and decreased in *sdo* overexpression strains, indicating a strong correlation between *sdo* genes and the tetrathionate intermediate pathway. Although the deletion or overexpression of *sdo* genes did not result in significant growth rate changes on Starkey-S^0^ medium, both strains showed altered transcriptions of genes in sox operon and heterodisulfide reductase complex operon (increased in *sdo* knockout strains and decreased in *sdo* overexpression strains)(Table 3). When tetrathionate was used as the sole energy, the overexpression of *sdo1* resulted in decreased transcriptions of most assayed genes (e.g. *hdrC2* and *dsrE2*), which was in agreement with the changes observed on S^0^. Δ*sdo2* increased the transcriptions of almost all assayed genes except *sdo1*, *dsrE1*, *sqr1* and *rhd3*, which was in agreement with the observed increased growth of this mutant grown on tetrathionate compared with the wild type (Fig 4B). Lastly, the OE-*sdo2* strain showed increased transcriptions of *sqr* and decreased transcription of *rhd*, indicating that *sdo2* may have a functional connection with SQR and Rhd (Table 3).

**Table 3:** Changes in the expression of sulfur oxidation genes in *A*.*caldus* MTH-04 in *sdo* knockout and *sdo* overexpression strains.

Gene	Locus	Gene description	Fold change (SD)^a^							
			S^0^			K_2_S_4_O_6_	S^0^		K_2_S_4_O_6_	
			Δ*sdo1*	Δ*sdo2*	Δ*sdo1&2*	Δ*sdo2*	OE-*sdo1*	OE-*sdo2*	OE-*sdo1*	OE-*sdo2*
Sox operon I										
*Sox X*-I	A5904_2486	cytochrome *c* class I	1.2±0.2	0.8±0.1	1.0±0.1	**17.7±8.1**	1.8±0.5	1.5±0.6	1.0±0.2	**2.6±0.5**
*Sox Y*-I	A5904_2487	sulfur covalently binding protein	1.0±0.3	0.9±0.2	1.1±0.3	**12.7±3.5**	**0.5±0.1**	**0.2±0.0**	1.1±0.3	**3.8±1.7**
*Sox Z*-I	A5904_2488	sulfur compound chelating protein	0.7±0.1	1.0±0.1	1.2±0.3	**10.2±0.5**	0.8±0.1	0.7±0.3	1.9±0.3	**6.3±2.2**
*Sox A*-I	A5904_2489	cytochrome *c* (diheme)	1.2±0.2	1.2±0.1	1.1±0.2	**4.5±0.4**	**0.3±0.2**	**0.3±0.2**	1.9±0.0	**6.5±3.1**
*Sox B*-I	A5904_2491	sulfate thiol esterase	**2.0±0.3**	1.7±0.4	**2.4±0.3**	**13.4±3.2**	1±0.2	0.6±0.3	0.9±0.0	**2.5±0.7**
Sox operon II										
*Sox Y*-II	A5904_2520	sulfur covalently binding protein	0.8±0.1	1.3±0.1	1.4±0.2	**13.3±0.5**	0.6±0.2	0.7±0.4	**0.4±0.2**	0.8±0.1
*Sox Z*-II	A5904_2521	sulfur compound chelating protein	0.7±0.1	1.3±0.1	1.5±0.3	**8.7±0.3**	**0.5±0.2**	**0.4±0.3**	**0.3±0.2**	**0.5±0.1**
*Sox B*-II	A5904_2522	sulfate thiol esterase	1.1±0.1	1.5±0.2	**2.5±0.3**	**22.7±0.9**	**0.4±0.1**	**0.3±0.2**	**0.3±0.1**	**0.5±0.1**
*Sox X*-II	A5904_2525	cytochrome *c* class I	0.9±0.1	**2.1±0.4**	**3.4±0.7**	**66.9±0.9**	1.1±0.3	1.0±0.5	**0.5±0.2**	0.9±0.3
*Sox A*-II	A5904_2526	cytochrome *c* (diheme)	0.8±0.0	1.6±0.2	1.9±0.6	**37.0±18.0**	0.7±0.2	**0.5±0.5**	**0.5±0.1**	1.0±0.1
Tetrathionate hydrolase operon										
*tetH*	A5904_1013	tetrathionate hydrolase	**2.7±0.2**	**3.5±0.7**	**2.3±0.5**	**8.2±1.4**	**0.4±0.1**	**0.3±0.2**	**0.4±0.0**	1.1±0.0
*tqo*	A5904_1014	thiosulfate quinone oxidoreductase	**2.4±0.1**	**3.1±0.4**	**2.4±0.4**	**11.9±0.4**	**0.3±0.1**	**0.2±0.2**	**0.3±0.0**	0.8±0.1
Sulfur dioxygenase										
*sdo1*	A5904_0421	sulfur dioxygenase	**0±0**	**0.4±0.1**	**0±0**	1.5±0.7	**2.7±0.5**	1.0±0.2	**6.7±0.6**	**9.3±4.3**
*sdo2*	A5904_0790	sulfur dioxygenase	1.0±0.3	**0±0**	**0±0**	**0±0**	**0.5±0.1**	**2.0±0.2**	1.7±1.5	**2.2±0.4**
Sulfide quinone reductase										
*sqr1*	A5904_1436	sulfide quinone reductase	1.3±0.1	0.8±0.1	1.5±0.4	1.7±0.8	**0.2±0.1**	**0.1±0.1**	1.7±0.2	**3.6±1.5**
*sqr2*	A5904_2678	sulfide quinone reductase	1.0±0.2	1.0±0.1	0.9±0.1	**2.5±0.9**	0.9±0.2	**0.4±0.0**	1.1±0.1	**2.7±0.4**
Sulfur transferase										
*dsrE1*	A5904_0418	DsrE-like protein	**0.5±0.2**	0.7±0.0	**0.3±0.1**	0.6±0.6	1.6±0.5	1.2±0.9	**0.3±0.1**	**0.2±0.0**
*rhd1*	A5904_0894	rhodanese (sulfur transferase)	1.4±0.3	0.9±0.1	1.2±0.2	**2.6±0.5**	**0.2±0.1**	**0.2±0.2**	**0.2±0.1**	**0.4±0.1**
*rhd2*	A5904_2860	rhodanese (sulfur transferase)	0.8±0.1	0.9±0.1	0.8±0.3	**4.9±0.3**	**0.5±0.1**	**0.4±0.4**	0.7±0.1	1.1±0.3
Heterodisulfide reductase complex operon										
*hdrC1*	A5904_1042	heterodisulfide reductase subunit C	0.7±0.0	0.9±0.1	1.1±0.2	**8.3±1.8**	**0.4±0.1**	**0.3±0.3**	0.8±0.1	**2.4±0.6**
*hdrB*	A5904_1043	heterodisulfide reductase subunit B	0.6±0.1	0.8±0.1	0.8±0.2	**3.0±0.1**	0.6±0.1	**0.2±0.0**	**0.5±0.3**	1.0±0.4
*hdrC2*	A5904_2472	heterodisulfide reductase subunit C	1.5±0.1	0.6±0.2	1.5±0.1	**2.2±0.9**	**0.3±0.1**	**0.4±0.2**	**0.4±0.1**	0.9±0.3
*dsrE2*	A5904_2473	DsrE (sulfur transferase)	**2.4±0.4**	0.9±0.1	**3.4±0.1**	**12.2±0.3**	**0.4±0.1**	**0.3±0.2**	**0.4±0.1**	1.0±0.0
*tusA*	A5904_2474	TusA (sulfur transferase)	1.4±0.4	1.6±0.2	1.0±0.1	**6.9±2.2**	1.6±0.4	1.6±0.8	1.0±0.5	1.7±0.7
*rhd3*	A5904_2475	rhodanese (sulfur transferase)	0.9±0.0	1.0±0.3	0.9±0.1	1.0±0.8	1.1±0.3	0.9±0.2	**0.1±0.0**	**0.2±0.1**

^a^ Δ*sdo1*, Δ*sdo2*, Δ*sdo1&2* represent the *sdo* knockout mutants; OE-*sdo1* and OE-*sdo2* represent the *sdo* overexpression strains of *A*. *caldus* MTH-04. Relative RNA transcript levels of these genes were determined by RT-qPCR analyses against the wild type for *sdo* knockout strains, or the control strain (wild type carrying pSDU1) for *sdo* overexpression strains. Fold Change ≧2 or Fold Change ≦0.5 and p< 0.05 were considered differentially expressed and highlighted in bold.

## Discussion

### Discovery and identification of SDOs in *A*. *caldus* MTH-04

**Fig 8:**
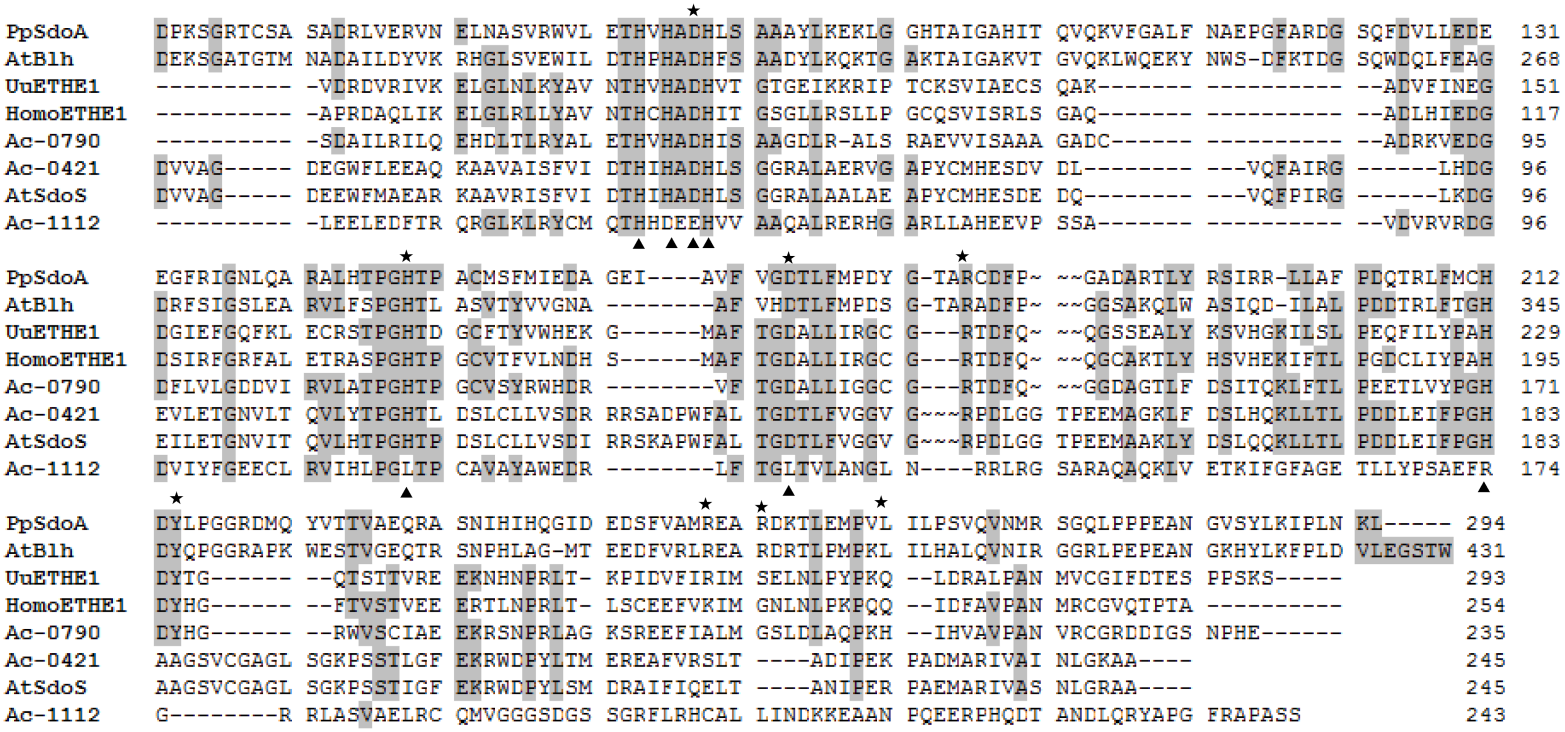
Sequence alignment of protein sequences homologous to SDO. The proteins encoded by A5904_0421, A5904_0790 and A5904_1112 from *A*. *caldus* MTH-04 are abbreviated to Ac-0421 (OAN04050.1), Ac-0790 (OAN03714.1) and Ac-1112 (OAN03376.1), respectively. The SDOs from *Acidithiobacillus thiooxidans*, *Agrobacterium tumefaciens*, *Homo sapiens*, *Pseudomonas putida* and *Urechis unicinctus* are abbreviated to At SdoS (WP_010641840.1), At Blh (Q8UAA9), Homo ETHE1 (NP_055112), Pp SdoA (ABQ76243) and Uu ETHE1 (AEV92813.1), respectively. Identical amino acids residues are highlighted in grey. The conserved residues in the metal binding site are marked under the row with triangle (▲). The conserved residues interacting with GSH are marked above the row with star (★).

Two new putative *sdo* genes (A5904_0421 and A5904_1112) were identified in the genome of *A*. *caldus* MTH-04 by conducting a BLASTP search with A5904_0790, however, only A5904_0421 had SDO activity. The proteins with SDO activity contain two conserved metal binding sites: site I consists of H113, H115, H169 and D188 and site II consists of D117, H118, H169 and H229 (in *U*. *unicinctus* ETHE1) [21]. Both of these metal binding sites were found in A5904_0421 and A5904_0790, respectively. However, A5904_1112 only had H113 of metal binding site I and H118 from metal binding site II (Fig 8). The metal binding sites play important roles in SDO activity in eukaryotic ETHE1s [14, 21, 25]. The absence of conserved metal binding sites in A5904_1112 may explain the lack of SDO activity of this gene. A conserved Y residue is present in the GSH binding site of *U*. *unicinctus* ETHE1 (Y231) and *P*. *putida* SdoA (Y214), however, a corresponding tyrosine residue was not found in A5904_0421 (Fig 8) [16, 21]. GSH was reported to activate the specific SDO activity of human ETHE1 [14]. Study of *U*. *unicinctus* ETHE1 showed that the lack of conserved residues in GSH binding site does not always affect the *K*m for GSSH, but results in reduced GSH binding capacity and lower SDO activity [21]. Thus the lack of conserved residues in GSH binding site may underlie the low SDO activity in A5904_0421 due to the low GSH binding capacity. In addition, we analyzed the sequences using ProtCompB, SignalP 4.1 and PSORTB v3.0, which predicted subcellular localization for the *A*. *caldus* SDOs. The SDO homologues do not contain signal peptides or transmembrane regions, suggesting that they might be cytoplasmic proteins, similar to other bacterial SDOs [11, 15]. However, whether the SDO proteins are localized to the cytoplasm or the periplasmic space requires further investigation.

### Distribution of SDOs in *Acidithiobacillus* spp and other sulfur oxidizing bacteria

Sequence analysis revealed that SDO homologues were widespread in *Acidithiobacillus* spp., and some *sdo* genes in *Acidithiobacillus* spp. are co-localized with other genes related to sulfur-metabolism. Both *sdo1* and *sdo2* are co-localized with other genes related to sulfur-metabolism in *A*. *caldus* MTH-04 (Fig 2) while the *sqr* gene (AFE_0267) is downstream of *sdo* (AFE_0269) in *A*. *ferrooxidans* ATCC 23270 as well [11, 26]. The co-localization of these genes on the chromosome suggests that they act cooperatively in sulfur metabolism. In addition, genes encoding transcriptional regulators are widely distributed upstream or downstream of *sdo* genes in *Acidithiobacillus* spp., indicating that *sdo* genes may be regulated at the transcriptional level. Transcriptional analysis revealed that the type of metabolic substrates that were available to *A*. *caldus* significantly influenced the expression of *sdo1*, which might be in accord with this hypothesis.

Our phylogenetic analysis of SDOs from other organisms identified a new SDO subgroup named SdoS, which predominantly contains SDOs from autotrophic sulfur-oxidizing bacteria. Previous studies on the structure of *P*. *putida* and *M*. *xanthus* SDOs supports the notion that SDOs may be classified into different subgroups by differences in the GS-moiety binding sites [16]. Blhs and SdoAs (also known as type II persulfide dioxygenase due to their use of glutathione persulfide as a substrate) have the following conserved residues in the GSH binding pocket: D78, H149, D170, R181, Y214, R250, R253 and L262. ETHE1 enzymes, also known as type I persulfide dioxygenase, do not contain R250, R253 and L262. Members of the SdoS group contain the conserved residues D78, H149, D170 and R181 (Fig 8). Differences in GS-moiety binding sites and different SDO activities suggest that there may be different mechanisms for substrate binding among different SDO subgroups. The presence of *sdo* gene duplications in different subgroups suggests that there may also be different mechanisms for sulfur oxidation. The identification of a new SDO subgroup may aid in the discovery of further *sdo* genes in other organisms. Further studies of *sdo* genes in these strains may shed light on the different types of SDO subgroups, and how they may be involved in sulfur oxidation through similar or complementary mechanisms.

## Sulfur dioxygenases in the sulfur metabolic network of *A*.*caldus*

SDOs from *Acidithiobacillus thiooxidans* and *A*. *ferrooxidans* are presumed to play a role in oxidizing extracellular elemental sulfur [9, 27]. However, the Δ*sdo1&2* strain grew well when elemental sulfur was used as the sole energy source (Fig 4A), and the *sdo* overexpression strains did not show increased growth rates. The results indicated that the SDO tested in this study does not play an essential role in the oxidation of extracellular elemental sulfur in *A*. *caldus*. Sulfur oxygenase reductase (SOR) is another elemental sulfur oxidation enzyme in *A*. *caldus*, which catalyzes the disproportionation of elemental sulfur to generate thiosulfate, sulfite, and sulfide. However, the *sor* gene in *A*. *caldus* MTH-04 has been lost during subcultivation in S^0^ media under laboratory conditions [22]. Deletion of *sdo1* significantly increased S^0^-oxidizing activity, indicating that the lack of *sdo1* may potentially stimulate other sulfur oxidation pathways. Deletion of *sdo* increased S^0^-oxidizing activity in *A*. *ferrooxidans* as well [11]. The high growth rates of the single and double *sdo* knockout strains in S^0^-media, and the high levels of elemental sulfur oxidation activity in these mutants, indicates that there are potentially undetected S^0^-oxidizing enzyme(s) in *A*. *caldus*.

**Fig 9:**
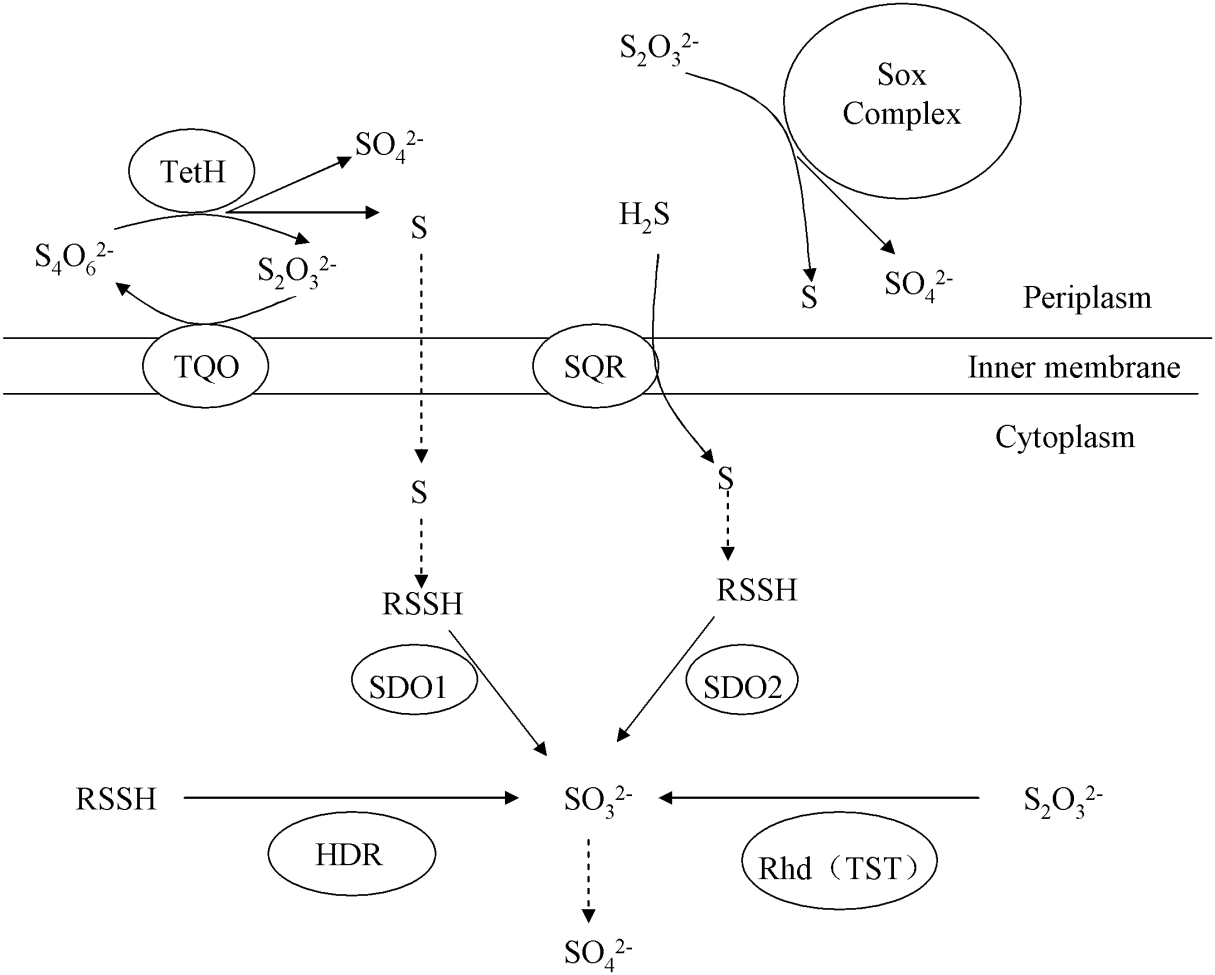
Proposal of the function of SDOs in sulfur oxidation in *A*. *caldus*. Abbreviations: TetH, tetrathionate hydrolase; TQO, thiosulfate-quinone oxidoreductase; Sox Complex, sulfur oxidation complex; SQR, sulfide:quinone oxidoreductase; Rhd (TST), rhodanese (also known as thiosulfate sulfurtransferase, TST); SDO, sulfur dioxygenase; HDR, heterodisulfide reductase. SDO oxidizes the elemental sulfur generated from the S_4_I and SQR pathways, sulfite is the product.

When tetrathionate was used as the sole energy source, the growth was much lower than that on sulfur (Fig 4). Similar low cell growth phenomena on tetrathionate measured by OD_600_ were also observed before [22, 28, 29]. Wild type *A*. *caldus* MTH-04 cells grown on tetrathionate until stationary phase yielded 3.56±0.44 g (dry wt.) cell material per mol tetrathionate, which was in agreement with the result reported by Shiers *et al*. [30]. The tetrathionate intermediate (S_4_I) pathway plays an important role in the metabolism of tetrathionate and thiosulfate in *Acidithiobacillus* spp. [26, 28, 31, 32, 33, 34]. The S_4_I pathway consists of a thiosulfate-quinone oxidoreductase (TQO), which oxidizes thiosulfate to tetrathionate, and a tetrathionate hydrolase (TetH) that hydrolyzes tetrathionate to thiosulfate, elemental sulfur and sulfate [35]. Deletion of *sdo1* caused complete inhibition of growth on tetrathionate, indicating that *sdo1* is necessary for *A*. *caldus* growth on tetrathionate. Deletion or overexpression of *sdo* genes resulted in the differential expression of *tetH*, *tqo*, and *dsrE2*. Previous studies have suggested that DsrE plays a role in thiosulfate transfer [36, 37]. This result was in agreement with the correlation between *sdo1* and the tetrathionate intermediate pathway. Based on the results of this study, we propose that *sdo1* plays a key role in the metabolism of TetH metabolites, for example, elemental sulfur (Fig 9). Due to the difficulties in analyzing intermediate metabolites in *A*. *caldus*, the specific mechanisms of the pathway will require further investigation.

Thiosulfate is a key intermediate in RISC metabolism in *Acidithiobacillus* spp, and is metabolized through the S_4_I pathway and the truncated sulfur oxidation (Sox) pathway [28, 31, 32]. The truncated Sox pathway consists of SoxYZ, SoxAX and SoxB. Deletion or overexpression of the *sdo* genes also resulted in the differential expression of genes involved in the Sox pathway (Table 3). Previous studies have suggested that SDO may substitute for the role of Sox(CD)_2_ in *A*. *caldus* [28, 31]. However, there was no corroborating evidence for this hypothesis from this study. The transcriptional changes that were observed in the Δ*sdo1* mutant may be due to a compensatory mechanism for the loss of *sdo1*.

Previous studies have established that SDO (ETHE1) catalyzes the oxidation of persulfides by interacting with SQR and Rhd [12, 13, 14, 38]. The transcriptional changes of *sqr* and *rhd* genes in the *sdo2* deletion or overexpression strains grown on different sulfur-substrates showed that *sdo2* may have a functional connection with SQR and Rhd (Table 3). Sulfide (H_2_S) is an important signaling molecule in human, animals and plants, and its accumulation can lead to cell damage [12, 13, 14]. The *sqr-sdo-rhd* pathway plays an important role in sulfur detoxification in mitochondria [12, 13, 14]. However, Hildebrandt *et al*. and Goubern *et al*. showed the possibility of getting energy by metabolizing sulfide (H_2_S) in mammalian, invertebrate and human cells, respectively [38, 39]. In *A*. *caldus*, sulfide (H_2_S) is an important intermediate metabolite of the sulfur metabolism and it can also be used as the energy source [5, 6]. In the reported sulfur metabolism models of *Acidithiobacillus* spp., sulfide (H_2_S) is suggested to be metabolized by SQR and produced electrons go to the quinol pool [26, 28, 31, 32, 33, 34, 40]. So, the *sqr-sdo-rhd* pathway may play a role in sulfur metabolism of *A*. *caldus*. Taken together, *sdo1* and *sdo2* may play different roles in *A*. *caldus*.

## Supporting information

S1 Fig Identification of the *sdo* knockout mutants of *A*. *caldus* MTH-04 by PCR. WT represent the wild type, Δ*sdo1*, Δ*sdo2*, Δ*sdo1&2* represent the *sdo* knockout mutants, respectively. 1, 2, 3, 4, 5, 6 represent the primer pairs 0421orfF-0421orfR, *sdo1*inF-*sdo1*inR, *sdo1*outF-*sdo1*outR, 0790orfF-0790orfR, *sdo2*inF-*sdo2*inR and *sdo2*outF-*sdo2*outR, respectively. The numbers on the right indicate the sizes of the fragments based on the molecular size maker (lane M).
(TIFF)

S2 Fig Purification of recombinant SDOs from *E*. *coli* BL21(DE3). The proteins are loaded on 10% (wt/vol) SDS-PAGE gel and stained with Coomassie Brilliant Blue R-250. 1, 2, 3 represent the purified recombinant proteins of A5904_0421, A5904_0790 and A5904_1112, respectively. M: Blue Plus^™^ II Protein Marker (TransGen Biotech).
(TIFF)

S1 Table Bacterial strains and plasmids used in this study. (DOC)

S2 Table Primers used in this study. (DOC)

S3 Table Predicted sizes of the fragments amplified by PCR with the primer pairs used in confirming the Δ*sdo* mutants. (DOC)

S4 Table Primers used in qPCR in this study. (DOC)

S5 Table Influence of inhibitors on SDO activities of purified recombinant A5904_0421. (DOC)
