# Supplementary figures and images for: Discovery of a new subgroup of sulfur dioxygenases and characterization of sulfur dioxygenases in the sulfur metabolic network of Acidithiobacillus caldus
Source: PLoS One. 2017 Sep 5;12(9):e0183668. doi: 10.1371/journal.pone.0183668 (PMC5584763; doi:10.1371/journal.pone.0183668)

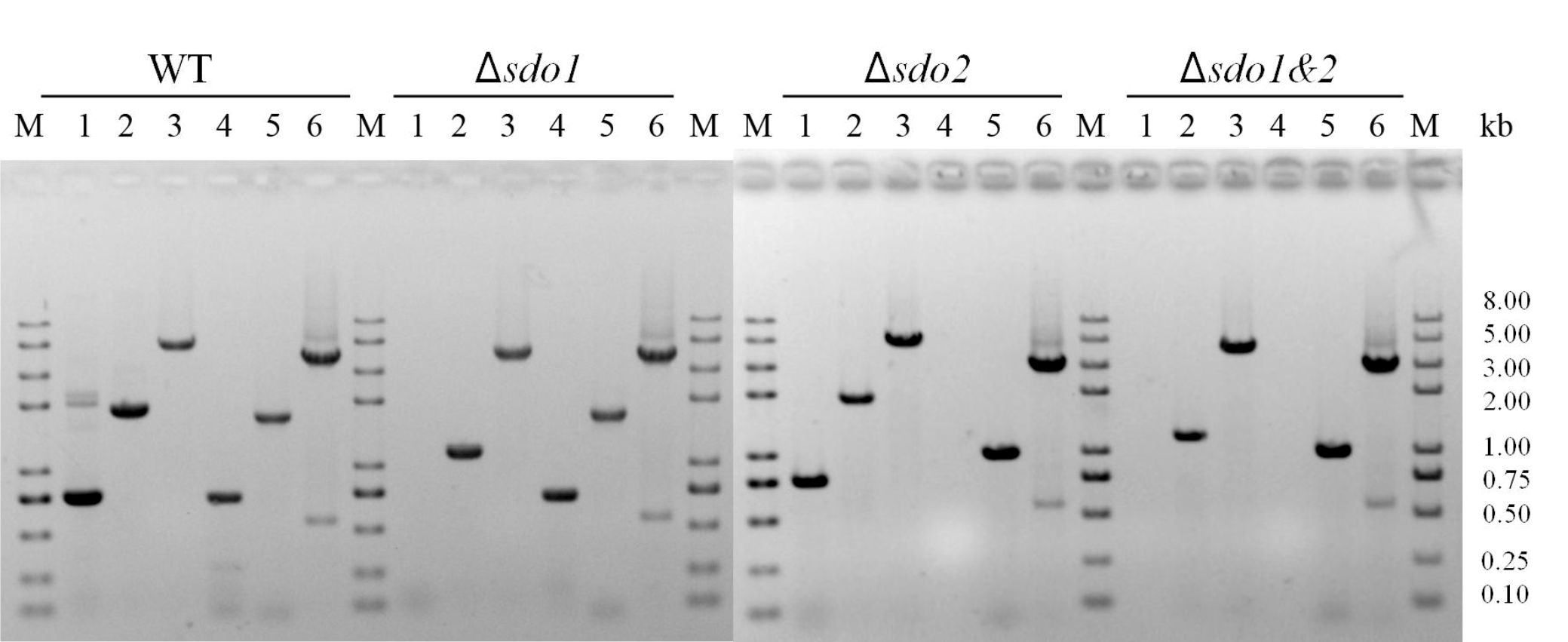

Supplement: S1 Fig — WT represent the wild type, Δsdo1, Δsdo2, Δsdo1&2 represent the sdo knockout mutants, respectively. 1, 2, 3, 4, 5, 6 represent the primer pairs 0421orfF-0421orfR, sdo1inF-sdo1inR, sdo1outF-sdo1outR, 0790orfF-0790orfR, sdo2inF-sdo2inR and sdo2outF-sdo2outR, respectively. The numbers on the right indicate the sizes of the fragments based on the molecular size maker (lane M). (TIFF) [file pone.0183668.s001.tiff]

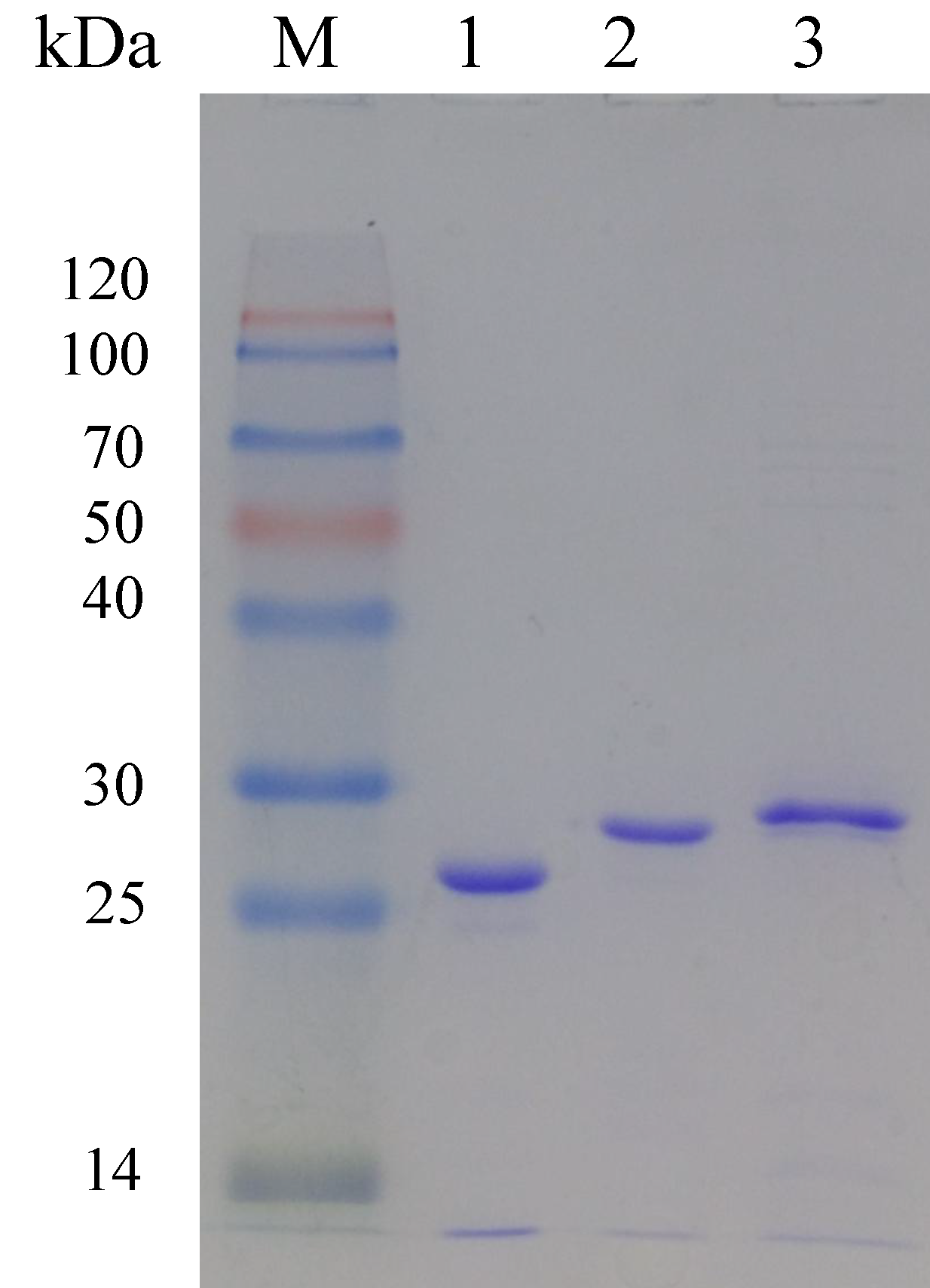

Supplement: S2 Fig — The proteins are loaded on 10% (wt/vol) SDS-PAGE gel and stained with Coomassie Brilliant Blue R-250. 1, 2, 3 represent the purified recombinant proteins of A5904_0421, A5904_0790 and A5904_1112, respectively. M: Blue Plus™ II Protein Marker (TransGen Biotech). (TIFF) [file pone.0183668.s002.tiff]
