# Supplementary material for: Discovery of a new subgroup of sulfur dioxygenases and characterization of sulfur dioxygenases in the sulfur metabolic network of Acidithiobacillus caldus
Source: PLoS One. 2017 Sep 5;12(9):e0183668. doi: 10.1371/journal.pone.0183668 (PMC5584763; doi:10.1371/journal.pone.0183668)
Supplement: S1 Table — (DOC) [file pone.0183668.s003.doc]

**S1 Table.** Bacterial strains and plasmids used in this study

| **Strain or plasmid** | **Genotype or description** | **Source or** |
| --- | --- | --- |
|  |  | **reference** |
| Strains |  |  |
| *Acidithiobacillus caldus* | | |
| MTH-04 | Wild type strain | (1) |
| Δ*sdo1* | MTH-04，ΔA5904_0421 | This study |
| Δ*sdo2* | MTH-04，ΔA5904_0790 | This study |
| Δ*sdo1&2* | MTH-04，ΔA5904_0421ΔA5904_0790 | This study |
| *Escherichia coli* | |  |
| JM109 | *recA1 endA1 gyrA96 thi-1 hsdR17supE44 relA1*Δ*(lac-proAB)/*F' | TaKaRa |
|  | [traD36proAB+ lacIqlacZΔM15] |  |
| BL21(DE3) | F^−^*dcm ompThsdS*(r_B_^−^m_B_^−^) *gal*λ(DE3) | Novagen |
| SM10 | Km^r^ *thi-1 thr leu tonA lacY supE recA*RP4-2-Tc::Mu | (2) |
| Plasmids |  |  |
| pET22b(+) | Ap^r^,ColE1 replicon; T7 promoter; *lacI*^q^ | Novagen |
| pET22b-0421 | pET22b(+) containing A5904_0421 | This study |
| pET22b-0790 | pET22b(+) containing A5904_0790 | This study |
| pET22b-1112 | pET22b(+) containing A5904_1112 | This study |
| pSDUDI | Ap^r^ Km^r^,*oriT*_RP4_,ColE1 replicon | (22) |
| pSDUDI-*sdo1* | pSDUDI carrying both homologous fragments of A5904_0421 | This study |
| pSDUDI-*sdo2* | pSDUDI carrying both homologous fragments of A5904_0790 | This study |
| pSDU1 | Cm^r^ IncQ mob^+^ | Our Lab |
| pSDU1-tac | pSDU1 containing *tac* promoter | Our Lab |
| pSDU1-I-Sce I | pSDU1 containing the I-Sce I gene | (22) |
| pSDU1-*sdo1* | pSDU1 containing A5904_0421 | This study |
| pSDU1-*sdo2* | pSDU1 containing A5904_0790 | This study |

**References**

1. Liu, Y., Qi, F., Lin, J., Tian, K., Yan, W. Isolation and phylogenetic analysis of a moderately thermophilic acidophilic sulfur oxidizing bacterium. *Acta Microbiol Sin*. 2004; 44(3):382–385.

2. Simon, R., Priefer, U., Pühler, A. A broad host range mobilization system for in vivo genetic engineering: transposon mutagenesis in gram negative bacteria. *Nat Biotechnol*. 1983; 1:784–791.
