# Supplementary material for: Discovery of a new subgroup of sulfur dioxygenases and characterization of sulfur dioxygenases in the sulfur metabolic network of Acidithiobacillus caldus
Source: PLoS One. 2017 Sep 5;12(9):e0183668. doi: 10.1371/journal.pone.0183668 (PMC5584763; doi:10.1371/journal.pone.0183668)
Supplement: S2 Table — (DOC) [file pone.0183668.s004.doc]

**S2 Table.** Primers used in this study

| **Primer** | **Sequence(5'-3')^a^** |
| --- | --- |
| 0421orfF | GGAATTCCATATGTTCTTCAAACAACGCGCAAGCG |
| 0421orfR | CCGCTCGAGTGCTGCTTTCCCCAGATTGATG |
| 0790orfF | GGAATTCCATATGTTATTCAAGCAGCTTTTTGACA |
| 0790orfR | CCGCTCGAGTTCATGTGGATTGCTCCCGATGTCG |
| 1112orfF | GGAATTCCATATGATCTTCAGACAGCTGTGTACCAAGGACAAAGTA |
| 1112orfR | CCGCTCGAGCGAAGAAGCCGGAGCCCGAAAGCCCGGCG |
| *sdo1*UF | CTAGTCTAGACCAGACCATCGCATAGACAC |
| *sdo1*UR | CCCAAGCTTATGAATAATGATACGGTTCGATATG |
| *sdo1*DF | CCCAAGCTTCACCTCCAGCATTCCGAAGTTAA |
| *sdo1*DR | ATAAGAATGCGGCCGCTCGGGTTGCTGATTGGAGTCTAT |
| *sdo2*UF | GCTCTAGATTTTCGCCCTCATCGTCATTCAGTTC |
| *sdo2*UR | GGAATTCCATATGATCCATCTTGCGTCCAGACTAACCG |
| *sdo2*DF | CCCAAGCTTATGAATGAATCCGACGATAACCCCCC |
| *sdo2*DR | ATAAGAATGCGGCCGCTGAATCCCACGCCTTTCCATCTCC |
| *sdo1*inF | TCACAAAGGAGGCGAGAA |
| *sdo1*inR | CCTGAGTGGTCGGCTTATT |
| *sdo1*outF | TGCTAAACCGTGAGACAAAG |
| *sdo1*outR | AGGATCTGGAAGGGTAAGAAG |
| *sdo2*inF | TGCACCGGGAGCAACAC |
| *sdo2*inR | GCTCGGGTAGTAAACGAAT |
| *sdo2*outF | TGCCTTGCATGTCCATTTCT |
| *sdo2*outR | CCTGCTTGGGTAACCAGTCC |
| *sdo1*F | CCCAAGCTTATGTTCTTCAAACAACGCGCAAGCG |
| *sdo1*R | TGCTCTAGATCAGTGGTGGTGGTGGTGGTGTGCTGCTTTCCCCAGATTGATGGCA |
| *sdo2*F | AACTGCAGATGTTATTCAAGCAGCTTTTTGACACCGAGAGC |
| *sdo2*R | TGCTCTAGATCAATGGTGATGGTGATGGTGTTCATGTGGATTGCTCCCGATG |
| pSDU1cxs | TTGTGCGGCTATGTCTGGC |
| pSDU1cxa | CATACACGGTGCCTGACTGC |

^a^ Artificial restriction sites are underlined
