# Supplementary material for: Discovery of a new subgroup of sulfur dioxygenases and characterization of sulfur dioxygenases in the sulfur metabolic network of Acidithiobacillus caldus
Source: PLoS One. 2017 Sep 5;12(9):e0183668. doi: 10.1371/journal.pone.0183668 (PMC5584763; doi:10.1371/journal.pone.0183668)
Supplement: S3 Table — (DOC) [file pone.0183668.s005.doc]

**S3 Table. Predicted sizes of the fragments amplified by PCR with the primer pairs used in confirming the Δ*sdo*** mutants.

| **Primer pairs** | **Templates and sizes of PCR products (bp)** | | | |
| --- | --- | --- | --- | --- |
|  | Wild type | Δ*sdo1* | Δ*sdo2* | Δ*sdo1&2* |
| 0421orfF/0421orfR | 738 | — | 738 | — |
| *sdo1*inF/*sdo1*inR | 1,906 | 1,168 | 1,906 | 1,168 |
| *sdo1*outF/*sdo1*outR | 4,846 | 4,108 | 4,846 | 4,108 |
| 0790orfF/0790orfR | 708 | 708 | — | — |
| *sdo2*inF/*sdo2*inR | 1,721 | 1,721 | 974 | 974 |
| *sdo2*outF/*sdo2*outR | 3,795 | 3,795 | 3,048 | 3,048 |

‘—’indicates no fragments could be amplified.
