# Supplementary material for: Discovery of a new subgroup of sulfur dioxygenases and characterization of sulfur dioxygenases in the sulfur metabolic network of Acidithiobacillus caldus
Source: PLoS One. 2017 Sep 5;12(9):e0183668. doi: 10.1371/journal.pone.0183668 (PMC5584763; doi:10.1371/journal.pone.0183668)
Supplement: S4 Table — (DOC) [file pone.0183668.s006.doc]

**S4 Table.** Primers used in qPCR in this study

| **Gene** | **Primer** | **Sequence(5'-3')** | **Product Size (bp)** |
| --- | --- | --- | --- |
| *sdo1* | 0421F | CTTGTCCTACTTCTACGGGTGC | 141 |
|  | 0421R | GCGTGAATGTGGGTGTCG |  |
| *sdo2* | 0790F | TTCACCTTGCCCGAAGAGAC | 179 |
|  | 0790R | ACCGCTACGTGGATGTGTTT |  |
| *soxX-I* | 2486F | TTCCCCTACGCCAATATGCC | 83 |
|  | 2486R | CAGAGGTAATCCGCCACCTG |  |
| *soxY-I* | 2487F | CACCCACCATTGCCGAAAAC | 200 |
|  | 2487R | GTTGTCGGTCTTGGCCATCT |  |
| *soxZ-I* | 2488F | GCCCACTTCATTCAGACCGT | 131 |
|  | 2488R | TTCAGTGTGCCGCTTTTCTC |  |
| *soxA-I* | 2489F | ATCCTGCCACATGGCTTACG | 180 |
|  | 2489R | GTACTTTCCAGGGGTTGGGG |  |
| *soxB-I* | 2491F | CGTTTCCGCCACGAAGAAAT | 137 |
|  | 2491R | CCGGCAGATCGAGCTTTTTG |  |
| *soxY-II* | 2520F | TGGCGAGCGCATTTTTCTTT | 115 |
|  | 2520R | AACTCACCCTTGTTCGTCCG |  |
| *soxZ-II* | 2521F | AAGCAAGGCAAGCTCATTCC | 129 |
|  | 2521R | GCGCATCTTGAAGGCAAGG |  |
| *soxB-II* | 2522F | CGTATCACAGACCTGCGTGT | 169 |
|  | 2522R | CCGGATTATCTCGGTGGTCG |  |
| *soxX-II* | 2525F | GAAGCTGGTGCAGTTTATTTACGA | 100 |
|  | 2525R | GCCAATCTGGTGATCCGTCA |  |
| *soxA-II* | 2526F | CTCTCCACTTTTGGGGCAGA | 136 |
|  | 2526R | ATCCGCGCTTTGCAGTTTTT |  |
| *tetH* | 1013F | CAACGGGGCCCGATCTATAC | 143 |
|  | 1013R | GTTGACCCAATCCCACGAGT |  |
| *tqo* | 1014F | AGGTTGCTCCGGATCCATTG | 113 |
|  | 1014R | TTTGTCATGCGAATTGGCCG |  |
| *sqr1* | 1436F | GACCCAAGACATTACCCGCA | 167 |
|  | 1436R | GTCGGTCACCACAAATCCCT |  |
| *sqr2* | 2678F | AGGTGGATGTCTTTGCCTACG | 156 |
|  | 2678R | TCGGTTCTTCCTTGAGACGG |  |
| *rhd1* | 0894F | CAGCCGGACGAATTTCTGCT | 161 |
|  | 0894R | CAACCCTCTATCTTGCCTCGT |  |
| *rhd2* | 2860F | GACCCATCGTCCGTAAGCTC | 209 |
|  | 2860R | GATGACGTGATGCTCCTGGT |  |
| *dsrE1* | 0418F | CTGGAGTTGGGACTGGAGG | 86 |
|  | 0418R | CTCTTAGCGACACCCTTTGG |  |
| *hdrC1* | 1042F | GGGTTTCTTCAAGCGTACCG | 129 |
|  | 1042R | TTGGGCCGAAACAGCGTATT |  |
| *hdrB* | 1043F | GCCGAAGTGGAATTTGGCAT | 126 |
|  | 1043R | ATCCATGGAATCGCTGACGTT |  |
| *hdrC2* | 2472F | GATCCAGCCCGACCACTT | 132 |
|  | 2472R | TTCAGCGACACCTCCCAC |  |
| *dsrE2* | 2473F | TCAGATGACGGTGGACCTCT | 116 |
|  | 2473R | TGAAAAGGGTGATGTCCGCT |  |
| *tusA* | 2474F | GTGGTCTCAACTGCCCCTTG | 118 |
|  | 2474R | GCTTCGAAATCCTTCACCGC |  |
| *rhd3* | 2475F | CCGGGCAGAATTTTCACCTC | 159 |
|  | 2475R | GGCCTCCGCTCAGACAATAG |  |
| *alaS* | *alaS*F | GACACCGACCTCTTCCAACC | 173 |
|  | *alaS*R | ACATAGCCACGCCGTTCATT |  |
