# Supplementary material for: Discovery of a new subgroup of sulfur dioxygenases and characterization of sulfur dioxygenases in the sulfur metabolic network of Acidithiobacillus caldus
Source: PLoS One. 2017 Sep 5;12(9):e0183668. doi: 10.1371/journal.pone.0183668 (PMC5584763; doi:10.1371/journal.pone.0183668)
Supplement: S5 Table — (DOC) [file pone.0183668.s007.doc]

**S5 Table. Influence** of inhibitors on SDO activities of purified recombinant A5904_0421.

| **Compound** | **Concentration (mM)** | **Remaining activity（%）** |
| --- | --- | --- |
| **None** | - | 100 |
| **Mg^2+^** | 1.0 | 95.38 |
| **Mn^2+^** | 1.0 | 61.94 |
| **Fe^3+^** | 1.0 | 47.95 |
| **Ni^2+^** | 1.0 | 0.04 |
| **Zn^2+^** | 1.0 | 47.44 |
| **Cu^2+^** | 1.0 | 0.05 |
| **Co^2+^** | 1.0 | 0.07 |
| **Hg^2+^** | 1.0 | 0.03 |
| **EDTA** | 1.0 | 0 |
| **DTT** | 0.05 | 77.42 |
| **NEM** | 1.0 | 0.11 |
